# Extraction, Structure–Activity Relationships, and Modification of Plant-Derived Polysaccharides: An Integrated Framework for Evidence-Based Method Selection and Bioactivity Optimization—A Review

**DOI:** 10.3390/ijms27198850

**Published:** 2026-10-03

**Authors:** Yuting He, Yu Xin, Xiwu Zhang, Di Han, Qichao Liang, Ling Kong, Yu Guan, Hui Sun, Chang Liu, Ying Han, Xijun Wang

**Affiliations:** 1 State Key Laboratory of Integration and Innovation of Classic Formula and Modern Chinese Medicine, National Chinmedomics Research Center, Metabolomics Laboratory, Department of Pharmaceutical Analysis, Heilongjiang University of Chinese Medicine, Heping Road 24, Harbin 150040, China; hyt1110821@163.com (Y.H.); 17804620737@163.com (Y.X.); 18846832189@126.com (D.H.);; 2 State Key Laboratory of Quality Research in Chinese Medicine, Macau University of Science and Technology, Avenida Wai Long, Macau, China

**Keywords:** polysaccharides, extraction, structure–activity relationship, biological activity, structural modification

## Abstract

Extensive research has been conducted on plant-derived polysaccharides, yet three interrelated challenges remain insufficiently explored: extraction methods are often selected without systematic validation; the structure–activity relationship (SAR) remains fragmented; and modification strategies are rarely designed on the basis of SAR knowledge. This article systematically summarizes three key aspects: extraction, SAR, and structural modification. We critically compare various extraction techniques and clarify that method selection requires consideration of source characteristics and application orientation. We also demonstrate that extraction conditions may affect structural integrity and, consequently, influence biological activity. Subsequently, we analyze SAR by examining molecular weight, monosaccharide composition, glycosidic linkages, uronic acid content, and chain conformation to identify structural determinants of biological functions. Finally, we review chemical modification strategies and illustrate how their efficacy depends on structural features initially determined by extraction. By integrating evidence from these three stages, this review offers a practical framework to support rational, evidence-based development of plant-derived polysaccharides for applications in medicine, food, and biomaterials.

## 1. Introduction

**Figure 1:**
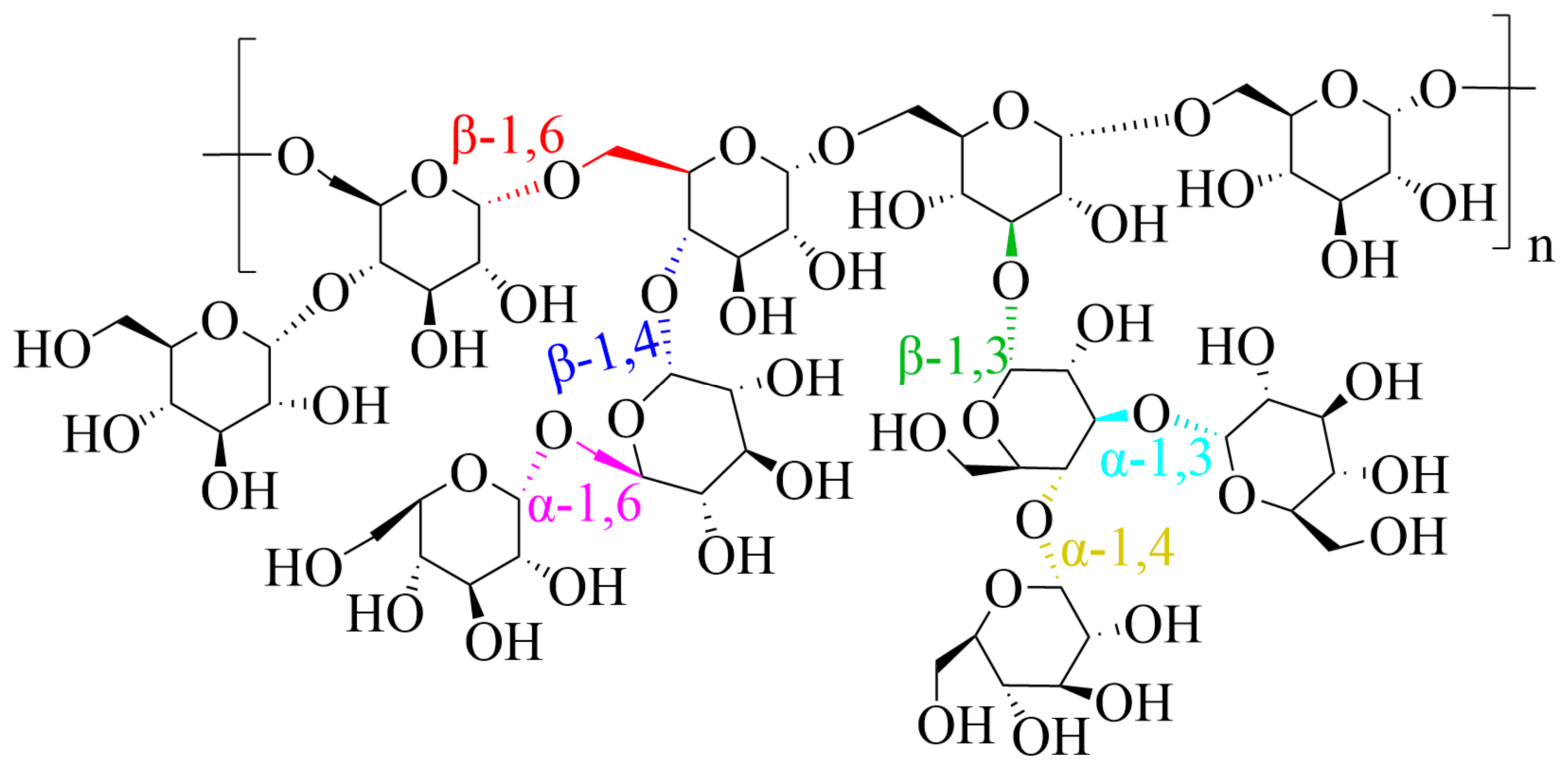
Structure of polysaccharide. A schematic diagram of the polysaccharide structure, which allows a view of the basic structure and sugar glycosidic linkage connection of the polysaccharide.

Polysaccharides are macromolecules comprising over ten monosaccharide residues linked via diverse glycosidic linkages (involving the α/β configurations of the 1 → 4, 1 → 3, and 1 → 6 bonds), which are abundant in plants, animals, and microorganisms, play crucial physiological roles in living systems, are generally considered to have low toxicity (although their toxicity may vary depending on source, purity, dose, molecular characteristics, and chemical modification) and possess multifaceted bioactivities [1,2] (Figure 1). These properties have positioned polysaccharides as viable candidates for therapeutic intervention and disease prophylaxis. However, the inherent complexity of polysaccharide structures poses significant challenges for extraction. Different extraction methods not only lead to variations in polysaccharide yield but may also influence their biological activity [3]. The traditional extraction methods exhibit limitations such as low extraction yield, long processing time, and large solvent consumption (like ethanol) [4]. To circumvent these limitations, microwave-assisted, enzymatic, and ultrasonic-assisted techniques have been developed successively, effectively improving the speed and yield of polysaccharide extraction [4,5]. Nevertheless, considering the simplicity of the experiment, the applicability of the factory and the cost maintenance, the water extraction/alcohol precipitation technique remains a commonly used polysaccharide extraction approach.

Plant polysaccharides have been reported to exhibit various biological activities, including antioxidant [6], anti-inflammatory [7], antimicrobial [8], anti-tumor [9], immunomodulatory [10], antiviral [11], anticoagulant [12,13], and hypoglycemic effects [14], which are closely related to their structural features, although the specific activity profile and potency vary depending on the source and structure. The monosaccharides that make up polysaccharides mainly include xylose, fructose, galactose, glucose, lactose, mannose, arabinose, and rhamnose, with some polysaccharides also containing galacturonic acid and glucuronic acid [15,16]. Differences in monosaccharide composition and proportions are associated with shifts in biological activity. The monosaccharide composition of acidic polysaccharides further encompasses uronic acid moieties, namely galacturonic acid and glucuronic acid. The uronic acid content also serves as a critical structural determinant influencing the bioactivity of polysaccharides [17]. Certain structurally complex polysaccharides suffer from limited water solubility and high viscosity, leading to poor absorbability and low bioavailability. Chemical modification offers a rational strategy to tune polysaccharide structure and biological function by targeting specific structural parameters, thereby enhancing their applicability in biomedicine and materials science. This review adopts an integrated, evidence-based perspective in synthesizing the literature across three interconnected dimensions, namely extraction, SAR, and structural modification, with the intention of providing a useful reference for the rational design and application of plant-derived polysaccharides in pharmaceutical, food, and biomaterial contexts.

This article is a narrative review. A literature search was conducted in Web of Science, PubMed and Google Scholar using keywords such as plant-derived polysaccharides, extraction, SAR, and structural modification. The search prioritized publications from the last five years, and for topics with limited recent literature, the search was extended to include earlier seminal works. The literature search was conducted in English, including peer-reviewed articles and academic dissertations.

Several recent reviews have addressed related topics. Zhang et al. [18] reviewed the relationship between extraction methods, physicochemical properties, structures, and biological activity of plant polysaccharides, but did not cover structural modification strategies, physiological stability, or safety evaluation. Huang & Huang [19] focused on extraction, separation, modification, and antioxidant activity. Yang et al. [20] discussed the effect of structure and preparation method on bioactivity of polysaccharides from plants and fungi. Li et al. [21] reviewed chemical modification and activity changes in land plant polysaccharides. While these reviews address related topics, they examine extraction, SAR, and modification as separate themes.

Unlike these reviews, the present work integrates extraction, SAR, and structural modification into a single framework unified by the concept of polysaccharide structure. This review focuses on plant-derived polysaccharides; microbial and animal polysaccharides are mentioned only when necessary for comparison or mechanistic illustration. In this work, polysaccharide structure links extraction, biological activity, and modification into a coherent framework. Table 1 compares this review with recent related reviews, highlighting the unique contribution of this work.

## 2. Polysaccharide Extraction Processes: Impacts of Extraction Approaches on Structural Characteristics

**Figure 2:**
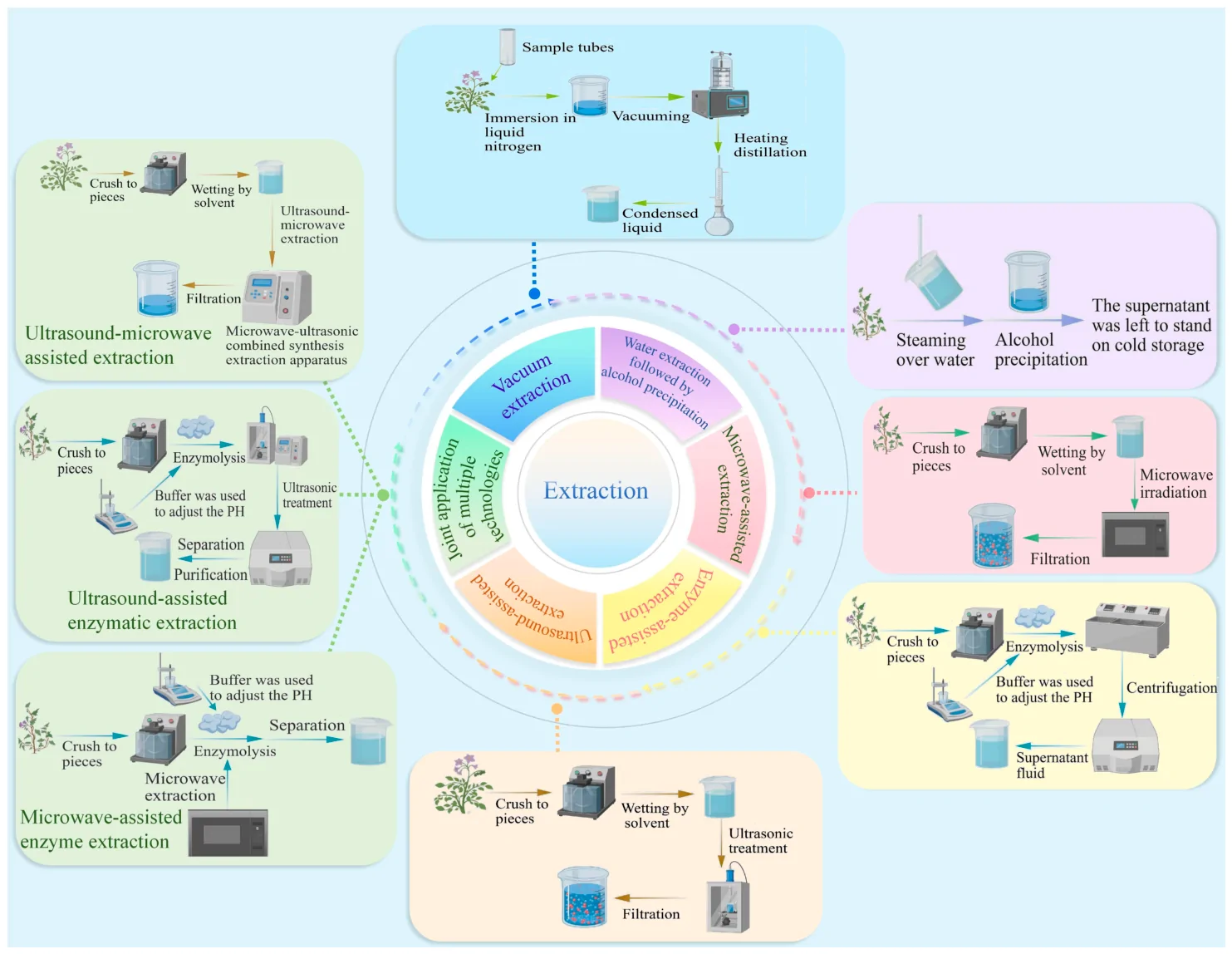
Method for extracting polysaccharides. The general flowchart of different extraction methods for polysaccharides can help us quickly understand the extraction techniques of polysaccharides and get a preliminary impression.

The extraction of polysaccharides is not only a separation process, but also a crucial step that has a profound impact on the subsequent study of their structural characteristics. Different extraction techniques disrupt plant cell walls through physical (such as heat, ultrasound, microwave) or biological (such as enzymatic) actions (Figure 2), which not only affects the extraction yield (Table 2), but also may influence the molecular weight, glycoside bonds, branching degree, and chain spatial configuration of the polysaccharides [24]. Therefore, the extraction protocol inevitably involves balancing productivity, structural preservation, and bioactivity. Based on this, we discussed the influence of the main extraction techniques on the correlation between polysaccharide structural features and activity.

### 2.1. Hot Water Extraction

The hot water extraction (HWE) method is the traditional and commonly employed technique for extracting polysaccharides. The principle is to dissolve the polysaccharides in hot water (typically at 60–100 °C), and then ethanol can be added for precipitation [31,32]. This method is operationally simple and economical, and it remains the preferred benchmark method for industrial production and laboratory research to this day.

Prolonged heating may result in the breakage of glycosidic linkages and shifts in the molecular weight of polysaccharides [33,34]. For instance, subcritical water extraction of ginger polysaccharides at 100–160 °C reduces their molecular weight from roughly 1080 kDa to about 310 kDa [35]. Meanwhile, the three-helix conformation, which is intimately associated with immunological activity, transitions into a random coil [35]. Studies show that at room temperature, prolonging extraction does not increase yield but reduces the average molecular weight of Japanese plum pectin (from 75,600 Da to 42,200 Da) [36]. However, extraction from boiling water (at 95 °C) shows a higher molecular weight (143 kDa), but there is a significant loss of arabinose (*p* < 0.05), which is likely to indicate the detachment of the RG-I domain [36]. This suggests that prolonged room-temperature extraction may still lower molecular weight, while heating may hydrolyze side chain glycosidic linkages and disrupt the polysaccharide structure. These findings indicate that although the high-temperature water extraction method achieves a high yield, it may impair the architectural integrity of polysaccharides at the higher-order level.

Nevertheless, through optimization of extraction conditions (temperature, duration, and solvent-to-solid ratio), the extraction performance and product yield can be enhanced. Under the optimized conditions (45 °C, 3 h, and a solid-to-liquid ratio of 1:15 g/mL), the yield of *Gossypium arboreum* L. seeds polysaccharide reached 8.67% [37]. After optimization (at 70 °C, with 70 W ultrasonic power, for 23 min, and a solid–liquid ratio of 1:10), the maximum extraction yield of *Cucurbita moschata* polysaccharides could reach 16.21% [38]. While modern auxiliary techniques are generally considered superior in yield and activity, the HWE still exhibits good bioactivity. For example, by employing various extraction methods, it was found that the HWE method yielded the highest polysaccharide content for all four species of *Ficus microcarpa* fruit, and also demonstrated strong in vitro antioxidant, anti-inflammatory and ECM enzyme inhibitory activities [39]. Additionally, *Amygdalus persica* L. flowers polysaccharide obtained via hot water extraction exhibited strong in vivo antioxidant effects in zebrafish models, significantly attenuating LPS-induced oxidative stress [40].

In conclusion, the stability of polysaccharides during the hot water extraction process may be closely related to their molecular structure. Polysaccharides containing furanose residues (such as arabinofuranosyl or galactofuranosyl) may be more susceptible to hydrolysis due to the inherently less stable five-membered ring, although this effect is primarily documented under acidic conditions [41,42], whereas pectic polysaccharides with high galacturonic acid content may undergo β-elimination under neutral-to-alkaline conditions at elevated temperatures [43]. Under more severe conditions, such as pressurized hot water or subcritical water extraction, even relatively stable polysaccharides may undergo molecular weight reduction [35]. These structure-dependent differences in thermal stability should be considered when selecting extraction conditions. Although HWE is a cost-effective and commonly used approach, high temperatures may induce a reduction in molecular weight and disruption of conformation. For polysaccharides that require the preservation of their higher-order structure (such as those with helix-dependent immunological activity), extraction conditions can be optimized, or alternative methods can be considered.

### 2.2. Physical Field-Assisted Extraction (Microwave/Ultrasound)

The physical field-assisted extraction can utilize the microwave thermal effect or ultrasonic cavitation effect to accelerate cell wall disruption and shorten the extraction time [44,45]. Moreover, these physical forces may also induce structural alterations to polysaccharide molecules.

Microwave-assisted extraction (MAE) utilizes the vibration of polar molecules in the microwave field to generate heat, accelerating the dissolution within cells [45]. MAE has been widely reported for its potential to improve extraction yield in terms of time, energy and solvent consumption; however, the actual realization of these advantages is highly contingent upon processing parameters and operational scale [46,47]. Under the optimized parameters, microwave-assisted extraction (550 W, 1:30 g/mL solid–liquid ratio, 70 °C, 6 min) of *Panax ginseng* polysaccharides under optimized conditions achieved a yield of 41.6 ± 0.09%, surpassing the 28.5 ± 1.62% obtained by HWE (90 °C, 5 h, 1:40 g/mL) [26]. Under the optimal conditions (liquid–solid ratio of 29:1 mL/g, reaction temperature of 74 °C for 15 min), MAE achieved a polysaccharide yield of 12.31% from *E. ulmoides* leaves, which was several times greater than that of the traditional hot reflux extraction method (5.62%) [48].

Ultrasound-assisted extraction (UAE) generates local high temperature and high pressure through cavitation, enhancing the solvent’s penetration ability [49]. Surin S et al. optimized the ultrasonic-assisted extraction conditions and found that the polysaccharide yield obtained by using UAE (150 W, solid–liquid ratio 1:20 *w*/*v*, 70 °C, 20 min) from defatted purple glutinous rice bran (*Oryza sativa* L.) was 4.3 ± 0.4%, which was higher than 0.8% obtained by HWE at 1:15 *w*/*v* and 90 °C for 2 h, and had certain antioxidant activity and the ability to chelate metal ions [50]. Similarly, Phosarith N et al. found that UAE (65% amplitude, 45 min) of crude polysaccharides from oat (*Avena sativa* L.) at a solid-to-liquid ratio of 1:20 *w*/*v* gave a yield of 82.72 ± 2.19%, approximately double that of HWE (90 °C, 35 min, 41.32 ± 0.98%), with the UAE extracts showing slightly better α-glucosidase and α-amylase inhibitory activities than those from HWE [44]. It has been demonstrated that physical-assisted extraction not only increases yield, but more importantly, enables the controllable reduction in polysaccharide molecular weight via the mechanical shearing action of ultrasound or microwave [51,52]. Extending the ultrasonic treatment time from 5 to 15 min significantly reduced the Mw of pomegranate peel pectin from 125.4 to 59.95 kDa (*p* < 0.05), whereas a further increase to 30 min raised it to 92.6 kDa [53]. Notably, the polysaccharide obtained after 30 min of treatment showed a more pronounced stimulatory effect on probiotic proliferation compared to the other samples [53].

Therefore, physical field-assisted extraction is not only an accelerator but also a molecular weight modulation tool, which may be used to specifically regulate the molecular weight of polysaccharides. For polysaccharides whose bioactivity depends on a defined molecular weight range, the molecular weight may be modulated by varying the intensity of ultrasound or microwave treatment, thereby enabling precise control.

### 2.3. Enzyme-Assisted Extraction

Enzyme-assisted extraction (EAE) utilizes cellulase, pectinase, papain, β-glucanase, and other enzymes to degrade cell wall components, thereby releasing polysaccharides under mild conditions (typically 40–60 °C) [54,55,56]. Compared to physical field-assisted extraction, an important feature of enzymatic extraction is its relatively mild operating conditions, which help reduce polysaccharide degradation during extraction and thereby better preserve their natural structure [57].

In terms of yield, the enzymatic extraction of some polysaccharides is superior to water extraction. Young Hulless Barley Leaves polysaccharides were extracted with a combination of xylanase and cellulase, yielding 22.63%, which was 5.5 times the yield of water extraction [30]. Using enzyme-assisted ultrasonic extraction, the polysaccharide yield from wild sotol (*Dasylirion wheeleri*) plants reached up to 77.59%, whereas conventional hot water extraction at 85 °C gave a yield of only 47.37% [58]. Furthermore, at a constant ultrasonic power, the extraction yield increased with increasing enzyme concentration within a certain range [58]. The enzyme extraction yield of Jabuticaba (*Plinia* sp.) peel pectin (22.06 ± 0.42% and 21.80 ± 2.25%) was higher than that of the traditional hot water extraction method (18.38 ± 0.35) [59].

From the perspective of structural preservation, enzymatic extraction involves mild conditions that may prevent damage to polysaccharide chains caused by high temperatures and intense physical shearing [60]. Moreover, by changing the extraction conditions, the molecular weight distribution may also be influenced. It has been reported that the selection of enzymatic extraction conditions affects the molecular weight profile of β-glucans [61]. For example, the molecular weight of barley bran β-glucans after three-step enzymatic purification can be as low as 2 × 10^4^ g·mol^−1^, while the molecular weight of the product obtained by simple treatment with α-amylase is 16 × 10^4^ g·mol^−1^ [61]. The use of β-glucanase can produce medium molecular weight (38,500–69,400 g/mol, 2 h, 10 nkat/g) or low molecular weight (11,800–22,600 g/mol, 4 h, 50 nkat/g) β-glucans from oats and barley [62]. EAE also affects the bioactivity of polysaccharides. The Dandelion *(Taraxacum mongolicum*) treated with 60 °C pectinase for 48 h and the non-enzyme hydrolyzed Dandelion were extracted in a 1:16 (*w*/*v*) solid–liquid ratio in an 80 °C water bath for 35 min, which resulted in the enzyme-extracted polysaccharides having a lower molecular weight, exhibiting stronger in vitro antioxidant activity, and more effectively reducing reactive oxygen species buildup, lipid peroxidation and cellular mortality in the zebrafish model [63]. Studies have shown that enzymatic extraction can also increase the molecular weight [64]. When different amounts of laccase were used for treatment, the average molecular weight of pectic polysaccharides from steam-dried sugar beet pulp increased from 97.1 kDa to 147 kDa, and as the molecular weight increased, the characteristic viscosity of this polysaccharide rose from 0.383 dL/g to 0.525 dL/g, while at the highest laccase concentration, the molecular weight reached 661 kDa and the viscosity reached 0.832 dL/g [64]. Additionally, by extracting polysaccharides from *P. edulis* peel through enzymatic methods, it has been clearly confirmed that the obtained polysaccharides have the highest integrity of the triple helix conformation and exhibit good antioxidant activity [65]. This indicates that for polysaccharides for which the SAR suggests that molecular weight or complete conformation is necessary for activity, enzymatic-assisted extraction may be the preferred method. Although enzymatic methods are more costly, this cost is reasonable in scenarios where the preservation of higher-order structure is required.

### 2.4. Vacuum Extraction and Multi-Technology Synergy

Vacuum extraction involves lowering the boiling temperature of the solvent under reduced pressure, allowing polysaccharides to be extracted at mild temperatures below those used in traditional hot water extraction (60–100 °C) [66,67]. At present, the vacuum extraction technology, as an independent extraction method, has not yet been reported in any relevant studies for the extraction of plant-derived polysaccharides. However, one study used vacuum-assisted extraction to isolate polysaccharides from *Lentinus edodes* stipe, achieving a yield of 4.28% at 62 °C and 0.08 MPa [66]. However, the yield of single vacuum extraction is limited. Vacuum technology is more often used as a complementary method. For example, in the extraction of marshmallow root (*Althaea officinalis* L.), the polysaccharide extraction yield of the OME (Omicron-Assisted Extraction) method was the highest (8.9%), but the OAVE (Vacuum-Assisted Omicron Heating) method demonstrated unique physicochemical properties, such as a higher oil retention capacity (12.3 g oil/g dry sample), which was almost twice that of the conventional Omicron extraction, and a maximum water retention capacity (82.5 g water/g dry sample); its outstanding water and oil retention capabilities serve an important function in the food industry (thickening and emulsification), food health (promoting intestinal health), and medicine and biological materials (drug sustained-release carriers) [68]. Zhang et al. found that the vacuum microwave drying method, which was carried out at a strength of 0.08 MPa for 75 min with intermittent operation and maintained at a temperature between 40 and 50 °C before extracting the *Rosa roxburghii* Tratt fruit polysaccharides (with a solid–liquid ratio of 1:20 and soaking at 90 °C for 2 h), exhibited excellent antioxidant and hypoglycemic biological activities [67].

The multi-technology collaborative extraction represents the current development direction of polysaccharide extraction techniques. A single extraction method often struggles to balance yield, structure retention, and cost. Physical field-assisted methods (ultrasound/microwave) have high extraction yield but may cause structural degradation, while enzyme-assisted methods are gentle in conditions but time-consuming and costly. Collaborative extraction, through the superposition of different physical and/or biological effects, is expected to achieve complementary advantages.

Ultrasonic-enzymatic-assisted extraction (UEAE) is among the most widely examined combined techniques. Ultrasound-induced cavitation can break the cell wall, increase the solubility of the solvent, and create a more favorable microenvironment for the action of enzymes, while the enzyme further decomposes the cell wall components under mild conditions, releasing bound polysaccharides [69]. Ultrasonic-assisted enzymatic extraction of orange peel pectin (solid-to-liquid ratio ≈ 1:19 *w*/*v*, 300 W ultrasonic power at 80 °C for 30 min, followed by enzymatic digestion at 50 °C for 4 h) afforded a yield of 26.9%, which was higher than that obtained by traditional acid extraction (solid-to-liquid ratio ≈ 1:20 *w*/*v*, 80 °C for 4 h; 22.1%), and the resulting pectin also exhibited a lower molecular weight [70].

The ultrasonic-microwave co-extraction method combines the advantages of both physical fields: the thermal effect of microwaves accelerates molecular movement, and the cavitation effect of ultrasound enhances solvent penetration. Studies have shown that microwave-ultrasonic-assisted extraction of polysaccharides from mangosteen peels achieved a yield of 18.4% under optimized conditions (66 min), and the extracted polysaccharides exhibited a structure similar to that of commercial pectin [71].

Microwave–enzyme coupling extraction utilizes microwave pre-treatment or auxiliary heating to enhance the enzymatic hydrolysis efficiency and shorten the total extraction time. During the extraction of polysaccharides from *Cynara cardunculus* tissues at a solid-to-liquid ratio of 1:100 g/mL with stirring at 70 °C for 5 min, microwave heating (250 W, 80 °C, 11 min) afforded a higher total carbohydrate content (95 g/100 g) compared to conventional convective heating (83 g/100 g), and subsequent buffer-enzyme treatment (protease and hemicellulase, 40 °C) further separated pectin- and inulin-rich fractions [72].

It is noteworthy that the significance of collaborative extraction goes beyond merely enhancing the yield, as by adjusting various technical parameters, such as ultrasonic/microwave intensity and enzyme types and ratios, it may also enable hierarchical and controllable modulation of the molecular weight and structural conformation of polysaccharides. For instance, within a certain range of ultrasonic power, changing the ultrasonic power may be used to purposefully prepare arabinoxylans fragments with different molecular weight ranges [73]. Additionally, choosing different enzyme types can selectively retain or remove specific glycosidic linkage connections [74]. This may upgrade collaborative extraction from a mere efficiency-enhancing tool to a platform for precise structural modulation. Therefore, collaborative extraction represents a paradigm shift from blind pursuit of high yield to integrated design of yield, structure, and activity.

### 2.5. Scalability and Industrial Applicability of Extraction Methods

In the laboratory extraction of polysaccharides, yield, structural integrity, and biological activity are the primary considerations, whereas industrial scale production involves additional constraints, such as scalability, cost-effectiveness, environmental impact, solvent recovery, and regulatory compliance [75,76]. An increasing number of studies emphasize that the extraction method should not only be evaluated for its yield and efficiency, but also for its industrial feasibility, which is a key factor in promoting the transition of laboratory achievements to industrialization [23].

#### 2.5.1. The Scalability of Polysaccharide Extraction

Scalability is a key factor in industrial applications. HWE is one of the traditional extraction processes. Owing to its simple equipment requirements and mature process parameters, hot water extraction offers high scalability and is therefore the method of choice in the food and medicinal fields, which typically employ large-capacity industrial-grade equipment, whereas auxiliary techniques such as MAE and UAE face numerous challenges when scaled up, including uneven heating, energy distribution issues, and equipment safety concerns in large-volume operations [77,78]. Similarly, although enzyme-assisted extraction is gentle and selective, it is also limited in terms of scale due to enzyme costs and stability.

Currently, there have been studies reporting a new and green extraction technology—the homogenization-assisted negative pressure cavitation extraction technology [79]. The homogenization-assisted negative pressure cavitation extraction (HNPCE) method was used to extract *Astragalus* polysaccharides, achieving a laboratory-scale yield of 16.74% under the optimal process conditions (homogenization time of 70 s, negative pressure of −0.068 MPa, temperature of 64.8 °C, solid–liquid ratio of 13.4, and processing time of 53 min), with the kinetic model showing about 85% recovery within the first 20 min, and a pilot-scale yield of 16.62% (3 kg, a 300-fold expansion) under the same conditions, basically consistent with the laboratory results [79]. Moreover, the HNPCE equipment has low investment costs, large raw material throughput, and low fixed costs per unit product, and although the homogenization pre-treatment increases a small amount of energy consumption, a short 70 s treatment can increase the yield from 12.19% to 15.42%, showing high cost-effectiveness, while using water as the sole solvent at a solid–liquid ratio of 1:13.4 with no organic solvents and recyclable water, making it environmentally friendly [79]. This suggests that this method has excellent scalability. Another international study demonstrated the optimization of a green subcritical water extraction process for extracting feruloylated arabinoxylan (AX) from wheat bran, in which a 33-fold scale pilot test was conducted, and although the total solid yield in the pilot test (32.6%) was significantly lower than that in the laboratory (46.7%), the cumulative yield of AX and the cumulative yield of ferulic acid (48.7%, 33.6%) were highly consistent with those in the laboratory (47.8%, 33.3%), verifying the scalability [80]. The pilot test used water as the solvent, which was recyclable and environmentally friendly, but the heating time (10 min) and the longer extraction time (60 min vs. 30 min) increased energy consumption, and although the pre-treatment for removing starch increased the process cost, it significantly improved the AX purity (with a solid–liquid ratio of 1:10 in the pilot test reaching 79.3%) and reduced the burden of subsequent purification, so the overall economic performance needed to be balanced among yield, purity, and energy consumption [80]. The above research indicates that the green extraction technology is not limited to the laboratory stage but also has great potential for industrial promotion and large-scale application and can be further applied in the industrial sector. Therefore, although the emerging green extraction technology faces limitations in terms of energy, temperature and output in industrial production, it is still possible to extract polysaccharides in large quantities and quickly by addressing the constraints on scalability or optimizing extraction conditions.

#### 2.5.2. Cost and Economic Feasibility

Cost considerations generally include raw materials, equipment, energy, labor, and waste disposal. Due to the relatively low capital investment and simple operation, hot water extraction remains a commonly chosen method in industry, although it does consume some energy costs for heating. Emerging green technologies may entail higher capital investment. For example, a techno-economic evaluation of enzymatic extraction of soluble β-glucans from oats and barley showed that, under a processing capacity of 100 kg/h, an enzyme dosage of 50 nkat β-glucanase/g bran, and an enzyme price of 32 €/kg, the production cost was 0.06–0.11 €/g, indicating that enzymatic polysaccharide extraction costs are influenced by multiple factors such as raw material prices, capital investment, and fixed costs, and require a comprehensive process-economic assessment [62]. Additionally, microwave extraction equipment is much more costly than conventional devices, yet it offers shorter processing times and lower solvent usage, which in turn reduces the expenses related to solvent recovery and waste disposal, thus decreasing the overall economic burden to some degree [4]. When using EAE for the industrial extraction of certain polysaccharides, in-depth process optimization is often required to achieve the optimal yield, and it may also be constrained by the cost of enzymes [54,81]. Therefore, in terms of economic feasibility, EAE may mainly focus on the extraction of high-value medicinal or food-grade polysaccharides. Techno-economic and environmental assessments indicate that when integrated functional extraction strategies are incorporated into cascading biorefinery systems capable of recovering by-products, the process may become economically competitive [82,83].

#### 2.5.3. Environmental Impact and Green Chemistry

In industrial polysaccharide production, environmental sustainability is receiving increasing attention. Although traditional HWE uses water as the solvent, it has high energy consumption and generates a large amount of liquid waste [84]. With the emergence of environmentally friendly extraction technologies such as microwave, ultrasonic, enzyme-assisted, and high-pressure liquid-phase extraction, these methods offer advantages including low solvent consumption, short processing times, and a low carbon footprint [85,86]. Furthermore, the use of green solvents, aqueous systems, and biodegradable enzymes further enhances the environmental friendliness of modern extraction methods [87]. Diribissakou et al. found that enzyme-assisted extraction can reduce carbon dioxide equivalent emissions and the E-factor, improve energy efficiency, and protect the environment [88]. Al-Akayleh et al. discovered that deep eutectic solvent (DES) extraction of β-glucan not only boosted the yield but also retained or even augmented the bioactivity, which is conducive to sustainable “green” extraction [89].

Studies have shown that quantitative data on the environmental performance of plant polysaccharide extraction exist but remain scattered across different raw materials and processes. For instance, ultrasonic-assisted extraction of hawk tea polysaccharides consumes 14.53 kWh/kg of energy, significantly lower than traditional extraction (25.34 kWh/kg), and emits 11.62 kg CO_2_/kg, only 57.33% of that of traditional hot water extraction [90]; the climate change impact of enzymatic extraction of beet pulp pectin is 39% lower than that of traditional methods, with the over-limit multiple of the safe operation space decreasing from 423 times to 238 times, and that in terms of regional phosphorus impact, enzymatic pectin will be below the absolute threshold in 2025, while traditional pectin has already exceeded the limit, with the main environmental burden of enzymatic pectin coming from the enzyme production process itself [82]. These data indicate that green extraction technology has clear environmental advantages in the field of plant polysaccharides; however, whether these environmental advantages can be translated into economic feasibility is still influenced by specific raw materials, scale, and process routes, and no widely accepted systematic comparison framework has yet emerged across various studies.

#### 2.5.4. Solvent Recovery and Waste Management

Solvent recovery and waste management are crucial economic and environmental considerations in industrial extraction. The conventional ethanol precipitation method consumes large volumes of ethanol, and without solvent recovery, this step adds considerably to the processing cost [91]. Therefore, during the experiment, ethanol is often recovered through distillation and reused; although the energy consumption is high, the technology is relatively mature and can achieve a high recovery rate [91]. Therefore, an efficient solvent recovery system may be adopted to save time and reduce costs.

Tang et al. developed a CO_2_-responsive ionic liquid (IL) for extracting *Rosa roxburghii* polysaccharides, which achieved a maximum yield of 253 mg/g under optimal conditions, could be recycled for at least five cycles without significant loss of efficiency, and featured reversible CO_2_-switchable properties that facilitated solvent recovery and reduced environmental impact [92]. Liu and Tan developed a DES-based aqueous two-phase system (ATPS) for *Astragalus membranaceus* polysaccharide extraction, which employed a choline chloride:urea (1:1) DES for solid–liquid extraction (yield: 141.11 mg/g) and ATPS for preliminary purification, enabling DES recycling and reuse with 97.85% extraction yield, indicating that this integrated method is simple, eco-friendly, and highly efficient for plant-derived polysaccharide extraction [93].

The downstream processes based on membranes, including ultrafiltration, dialysis, and reverse osmosis, are attracting attention due to their ability to purify polysaccharides while reducing solvent usage [94,95]. A study employed ultrafiltration and dialysis to recover galactoglucomannan from spruce sawdust [96]. This water-based process replaced organic solvents, with ultrafiltration increasing purity from 48% to 63% with an 88% retention rate, and dialysis removing monosaccharide impurities, thereby reducing organic solvent usage [96]. Additionally, pressurized hot water extraction of galactoglucomannan from low-value sawdust achieved ~70% recovery, enabling waste utilization [96].

#### 2.5.5. Summary of Industrial Applications

In summary, industrial scale polysaccharide extraction requires simultaneous consideration of yield, quality, cost, and sustainability. HWE remains the most prevalent method for large-scale manufacturing due to its proven protocols and cost advantages, whereas emerging green technologies offer environmental benefits but face challenges in scalability and cost. Integrating extraction with downstream processing and by-product utilization is key to promoting a green, sustainable, and profitable polysaccharide industry.

### 2.6. Summary: From Extraction Science to Industrial Application

Despite the general trends described above, contradictory findings exist regarding the relationship between extraction method and polysaccharide properties. For instance, ultrasonic treatment has been reported to affect the molecular weight of polysaccharides depending on power, duration, and polymer concentration [53,73]. Similarly, enzymatic extraction has been shown to potentially reduce molecular weight depending on the type of enzyme used and the duration of the treatment [61]. However, through the cross-linking effect mediated by laccase, the molecular weight can also be increased [64]. These discrepancies may arise from differences in raw material composition, equipment parameters, solid–liquid ratio, and methods used for structural characterization. Such contradictions highlight the need for standardized reporting of extraction conditions and structural outcomes, without which cross-study comparison remains difficult.

It is important to note that a high extraction yield does not necessarily indicate a superior method. Method selection should be evaluated across multiple dimensions, including purity, molecular weight preservation, biological activity, energy consumption, solvent usage, cost, and scalability. For example, microwave-assisted extraction may achieve higher yields than hot water extraction, but may also affect molecular weight and structural features, which could influence bioactivity [26,48]. Enzyme-assisted extraction preserves structural integrity but is limited by enzyme cost and processing time [81]. Hot water extraction remains the most scalable and cost-effective option, but may not be suitable for polysaccharides whose activity depends on high-molecular-weight or triple-helix conformations. Therefore, the optimal extraction method depends on the target polysaccharide, the desired structural features, and the intended application, rather than yield alone.

From an industrial perspective, HWE remains the most prevalent method for large-scale manufacturing due to its proven protocols and cost advantages. Emerging green technologies offer environmental benefits but face challenges in scalability and cost, and the scalability of combined technology strategies still needs further verification. Integrating extraction with downstream processing and by-product utilization is key to promoting a green, sustainable, and profitable polysaccharide industry.

Based on the above analysis, we propose an integrated framework (Table 3). Different extraction methods may shape the biological activity spectrum of polysaccharides by influencing structural attributes including molecular weight, chain conformation, and branching density. The selection of extraction methods is not a neutral technical decision but may shape the structural identity and potential activity spectrum of polysaccharides. The differences in the bioactivities of polysaccharides are associated with the structural variations induced by different extraction methods.

## 3. Advances in Understanding Polysaccharide Structure-Biological Activity Correlations

Rather than being dictated by a single structural factor, the bioactivity of polysaccharides reflects the integrated action of multiple structural elements, comprising molecular weight, monosaccharide composition, patterns of glycosidic linkages, branching degree, uronic acid content, and chain morphology. Currently, research on SAR is transitioning from a one-factor search paradigm toward a multi-factor interaction network analysis. This shift may hold direct practical implications for how extraction methods are selected and how structural modification strategies are designed.

### 3.1. Heterogeneity of Plant Polysaccharide Extracts

Before discussing structure–activity relationships, it is important to recognize that most plant-derived polysaccharide extracts are heterogeneous mixtures rather than pure, uniform compounds. Crude polysaccharide extracts typically contain co-extracted proteins, polyphenols, and other small-molecule contaminants, and even after purification, polysaccharide fractions often exhibit broad molecular-weight distributions and may contain both neutral and acidic polysaccharide components.

These heterogeneities can influence the observed bioactivity, for example, protein contaminants may contribute to immunomodulatory effects, polyphenols may contribute to antioxidant activity, and the coexistence of neutral and acidic fractions may lead to synergistic or masking effects [97,98]. Studies have reported an association between the antioxidant activity of arabinoxylans and their phenolic substance content [73]. Other studies have also shown that although the antioxidant activity of pectin is attributed to the hydroxyl and carboxyl groups in its monosaccharide units, the phenolic compounds contained therein do participate to some extent in DPPH free radical scavenging [53]. And further studies have shown that the polysaccharides obtained through methods such as heating reflux extraction, EAE, and MAE all contain a small amount of protein and polyphenol impurities, which may adversely affect the purity of the polysaccharides, and moreover, the antioxidant activity of the polysaccharides is related to their structure and may also be affected by proteins or polyphenols [28]. Furthermore, the degree of heterogeneity varies with the extraction and purification method, which complicates cross-study comparison. However, most published studies attribute the observed bioactivity to the polysaccharide fraction itself, and in some cases, purification has been used to confirm this, although trace contaminants may remain [99,100]. Unless otherwise specified, the bioactivities discussed in this review refer to those of the polysaccharide fractions as reported in the original literature. The potential contribution of co-extracted contaminants should nonetheless be kept in mind when interpreting individual studies.

### 3.2. The Connection Between Molecular Weight and Biological Function

Molecular weight constitutes one of the most fundamental structural features of polysaccharides, affecting their physicochemical properties (including solubility and chain conformation) and biological activity [101]. However, the correlation between molecular weight and bioactivity is not simply linear; rather, it emerges from the interplay of multiple factors, including the polysaccharide chain conformation, branching degree, and target sites [102]. Although high molecular weight facilitates the formation of triple-helical higher-order structures, excessive molecular size increases solution viscosity and reduces solubility, thereby hindering cellular uptake and cell-surface receptor accessibility [103,104,105]; while a low molecular weight allows for easier solubility in water and penetration of tissue barriers, an excessively low degree of polymerization may result in a lack of sufficient ordered domains, making it difficult for the polysaccharide to be effectively recognized or bound by immune receptors [103,104]. Studies have indicated that the molecular weight ranges of polysaccharides with superior bioactivity vary across sources, owing to differences in their overall structural characteristics. Moreover, different components obtained from the same polysaccharide also vary in terms of molecular weight and bioactivity (Table 4). The advantages of low molecular weight polysaccharides in antioxidant and immunomodulatory activities have been widely supported by literature [106]. For instance, the 25.934 kDa component of the *Artemisia argyi* polysaccharides exhibited stronger in chemical-assay scavenging activity against DPPH, hydroxyl, and superoxide anion free radicals compared to its 89.682 kDa high-molecular-weight polysaccharide [107]. The *Cuminum cyminum* polysaccharides CCP2 with a lower molecular weight (2.03 × 10^4^ Da) exhibited the strongest ability to induce NO production in RAW264.7 cells studies and the best effect on promoting cell proliferation, while also activating both RAW264.7 and NK-92 immune cells through the NF-κB and MAPKs signaling pathways [108]; in cell studies, the low molecular weight (16.96 kDa) fraction of litchi polysaccharides exhibited stronger immunomodulatory activity than the higher-molecular-weight (378.67 kDa) fraction, and in the microbiological assays, it also promoted probiotic proliferation more effectively [109].

However, lower molecular weight does not always correlate with greater activity. The CPPS-II component with medium molecular weight (9.8 × 10^5^ Da) of *Codonopsis pilosula* polysaccharides (CPPS) has stronger immunomodulatory ability and relatively stronger anti-tumor activity in both cell studies and animal experiments [110]; three polysaccharide components (NJBTF1 (90 kDa), NJBTF2 (31 kDa), and NJBTF3 (19 kDa)) isolated from *Cucurbita moschata* displayed varying biological activities in immunogenic assays, with NJBTF1, the highest molecular weight fraction, exhibiting the strongest activity [111]; in chemical assays, among the four groups of *Sagittaria sagittifolia* L. polysaccharides with different molecular weights, the high-molecular-weight group (294.9 kDa) exhibited stronger scavenging ability against DPPH, ABTS and hydroxyl radicals, and its antioxidant activity was superior to that of the other groups (52.0/229.4/230.6 kDa) [112]. These studies also indicate that molecular weight may not be the sole or dominant factor influencing activity, but rather acts together with fine structure and target sites, and polysaccharides from different plant sources may exhibit different activities. Due to disparities in sugar composition, glycosidic linkage types, branching degree, and higher-order structures, different polysaccharides may exhibit distinct binding patterns to receptors and signaling pathways; therefore, their optimal molecular weight ranges also vary depending on the specific polysaccharide system.

Based on this understanding, choosing an appropriate initial extraction method is of great importance. As an important process for modulating the molecular weight distribution and structural features of polysaccharides, this method should be rationally designed based on the expected activity of the target product, rather than randomly selecting an extraction method and proceeding directly with the research.

### 3.3. Monosaccharide Composition and Proportion: Key Parameters Influencing the Structural Features and Bioactivity of Polysaccharides

Monosaccharides are the building blocks of polysaccharides, modulating the electrostatic charge, reactive groups, and biological activity exhibited by polysaccharides. Their composition and proportion serve as the foundation for investigating the links between the structural attributes, physicochemical traits, and physiological functions of polysaccharides [101,113]. The monosaccharides that make up polysaccharides primarily include fucose (Fuc), arabinose (Ara), fructose (Fru), galactose (Gal), mannose (Man), rhamnose (Rha), glucose (Glc), ribose, and xylose (Xyl), some polysaccharides also contain uronic acid and glycosamine [114].

Monosaccharide types supply the essential chemical framework for polysaccharide activity, and the molar ratio of these monosaccharides is closely related to the biological activity [115]. Three anti-inflammatory polysaccharide components from the roots of *Isatis indigotica* Fort (RIP-A1, RIP-B1, RIP-B2) all contain Ara and Gal, but with distinct molar ratios of 2.19:1.70, 5.00:5.58, and 3.30:1.00, respectively; in cell studies, the component with the most balanced ratio (RIP-A1) shows the greatest anti-inflammatory potency [116]. Additionally, in chemical assays and animal experiments, a higher D-mannose/D-glucose ratio may influence the hypolipidemic activity of *Dendrobium officinale* polysaccharides [117]. Thus, the bioactivity of polysaccharides is influenced by both the identity and the relative abundance of their constituent monosaccharides.

It is noteworthy that the same source of monosaccharide can exert diametrically opposite effects, which might be related to the specific monosaccharide species. For instance, in chemical assays, the galactose-containing fraction LLp-1b from *Ligustrum lucidum* flower polysaccharides (LLp) demonstrated procoagulant activity, whereas two other fractions (LLp-1a and LLp-3) which contain galactose, exhibit anticoagulant activity; notably, the latter two also contain rhamnose and xylose [118].

Thus, the function of a given monosaccharide may depend on its structural context and cannot be understood in isolation from the overall polysaccharide architecture. This realization underscores the research importance of preserving the monosaccharide composition and ratios of highly active polysaccharides during extraction. In addition, extraction methods differ in how well they retain neutral sugars versus glucuronic acids, which may directly influence the resulting product’s activity spectrum.

### 3.4. Sugar Glycosidic Linkages and Branching Degree: Important Structural Parameters for Activity Recognition

Various monosaccharides in polysaccharides are linked via glycosidic linkages to form different sugar chains and branches, while the type and linkage mode of these bonds, together with the degree and structure of branching, exert a significant influence on the functional activity of polysaccharides [119]. The primary types of glycosidic linkages in polysaccharides are the α and β types. Examples of typical glycosidic linkage types include α-(1 → 4/6) and β-(1 → 3/4/6) [119]. Polysaccharides’ structural heterogeneity and diversity arise from the numerous ways in which these glycosidic linkages can be combined and connected. Conversely, when the chain structure of polysaccharides changes, the glycosidic linkages also change. For example, in the process of branched chain destruction, when the branching degree decreases, the α-1,6-glycosidic linkage is easily destroyed [101,120,121,122].

**Figure 3:**
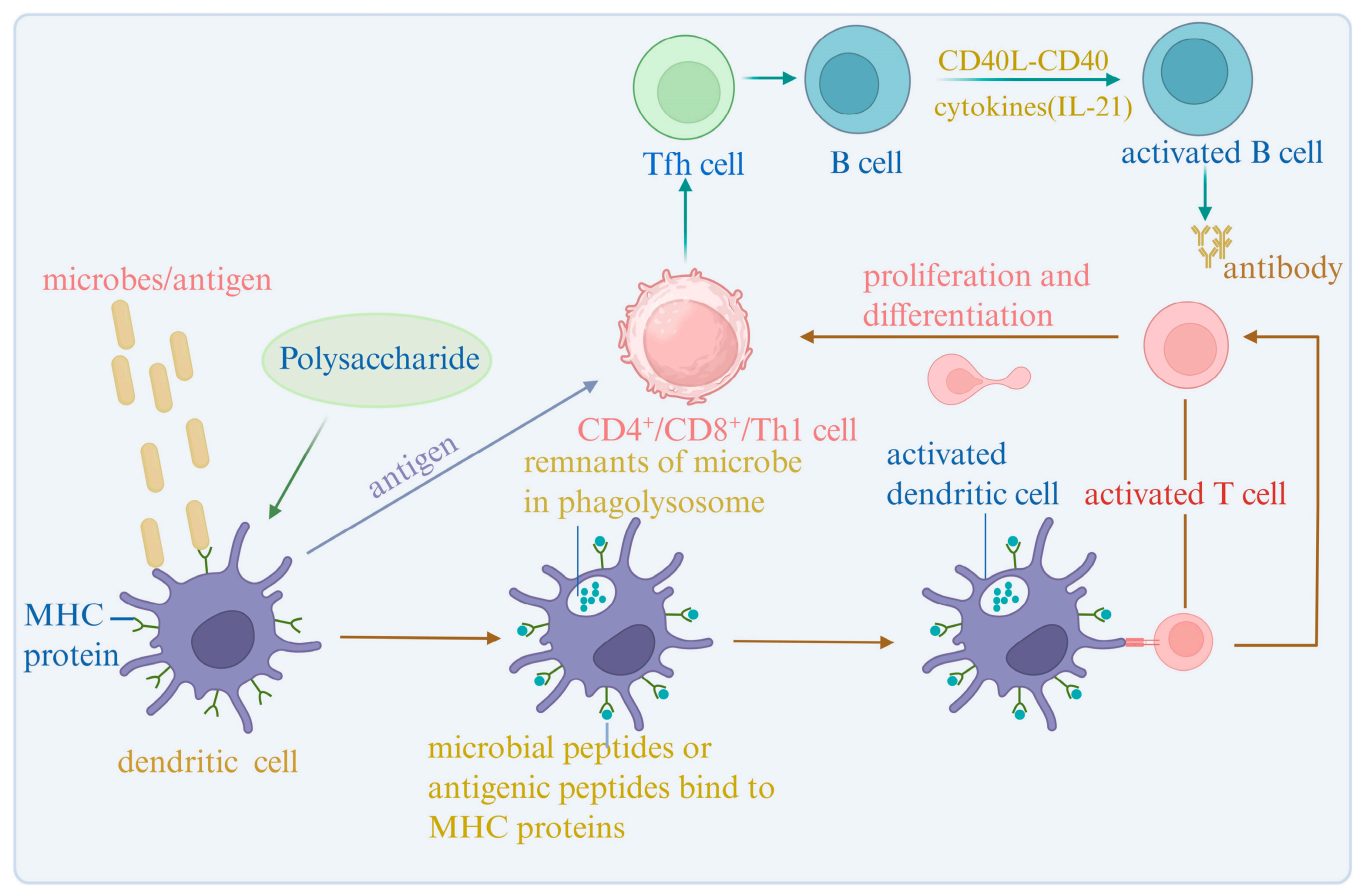
The immunomodulatory effect of polysaccharides. This figure demonstrates that polysaccharides may enhance the body’s ability to resist microbial invasions by influencing both cellular immunity and humoral immunity.

β-(1 → 3) and β-(1 → 6) glycosidic linkages have been proposed to contribute to the bioactivities of polysaccharides, such as immunomodulation (Figure 3), antioxidant activity, and anti-inflammatory effects [123]. In *Isatis indigotica* polysaccharides, the β-(1 → 3,6)-galactan skeletal chain may mediate immune-enhancing activity, whereas the 1,3,5-arabinofuranosyl branches may be associated with anti-inflammatory effects [124]. Moreover, the α-(1 → 5)-Araf branches seem to enhance humoral and cellular immunity, conferring antiviral and anti-inflammatory properties [124]. Additional evidence indicates that side chains significantly contribute to the bioactivity of polysaccharides. In the in vivo experiments of pectin polysaccharides, orange pectin with abundant neutral sugar side chains exhibited significantly stronger immunomodulatory and intestinal protection activities, while the activity of citrus pectin with a large number of missing side chains was significantly weakened in animal experiments [125]. Likewise, in animal studies, neutral multi-branched fractions of *Lycii fructus* polysaccharides showed superior anti-aging properties relative to acidic polysaccharides [126].

Glycosidic linkage patterns and branching levels remain essential structural markers for receptor recognition of polysaccharides. Research has shown that oat β-glucan can activate the Dectin-1 receptor and induce immune stimulation in human dendritic cells, which is significantly enhanced after enzymatic hydrolysis with endogenous glucanase. This enhanced immune response is attributed to the increased activation of the Dectin-1 receptor, which is contingent upon the exposure of β-(1 → 3)-glycosidic linkages and the reduction in the size of β-glucan particles after enzymatic hydrolysis [127]. Therefore, the presence of certain specific glycosidic linkages may affect the bioactivity of polysaccharides. Moreover, specific galactose-containing sugar chain segments within pectic heteropolysaccharides demonstrate strong binding affinity for TLR4, thereby selectively triggering TLR4-mediated activation of macrophage phenotypes [128]. Consequently, the glycosidic linkage mode and branching ratio together are associated with a molecular fingerprint for receptor recognition. Different branching patterns may influence receptor binding affinity and the ability to activate bioactivity.

In conclusion, glycosidic linkage patterns and branching architecture jointly influence polysaccharide bioactivity, with branching playing an especially prominent role. High branch density may induce spatial steric hindrance, inhibit intermolecular hydrogen bonding, and thereby enhance water solubility, whereas low branch density may favor the formation of dense helical structures or high-viscosity gels, consequently reducing water solubility [129]. Thus, branching degree may be an important factor influencing bioactivity and water solubility.

### 3.5. Uronic Acid Content: The Active Code of Pectin-Type Polysaccharides

Pectin polysaccharides extracted from natural plants are generally composed of four main domains: rhamnogalacturonan-I (RG-I), homogalacturonan (HG), xylogalacturonan (XGA), and rhamnogalacturonan-II (RG-II) [130]. They exhibit various pharmacological properties, including immunomodulation, inflammation-reducing, hypoglycemic, bactericidal, radical-scavenging, and anti-tumor effects [131].

Research has revealed a dose-dependent positive correlation between the sugar aldehyde content of certain polysaccharides and their biological activity [132]. After exposure of black garlic polysaccharides to sulfuric acid at various intensities, uronic acid content positively correlated with tyrosinase inhibition in an in vitro chemical assay [133]. Additionally, in cell studies, galacturonic acid (GalA) content is positively associated with the antioxidant activity of *Lycii fructus* polysaccharides [126]. The role of uronic acid composition may extend beyond modulating protein binding affinity and bioactivity. It may also act as a direct active ligand for specific membrane receptors, thus participating importantly in signal transduction. In chemical assays, during the simulated digestion and fermentation process, the enrichment of uronic acid (especially galacturonic acid) enhances the bile acid binding ability of kiwi fruit polysaccharides, while a decrease in its content leads to a weakening of the activity [132]. A study on pectic heteropolysaccharides also indicates that polysaccharides obtained from plant cell walls can trigger macrophage responses by binding to the TLR4 receptor, suggesting that plant-derived polysaccharides may interact with the TLR4/CD14 receptor system [128].

Nevertheless, research into glucuronic acid has so far been confined primarily to establishing correlations. The detailed molecular mechanisms, including the precise mode of interaction between glucuronic acid and its receptors, are still largely unclear.

### 3.6. Chain Conformation: Functional Transition from Primary Structure to Higher-Level Structure

Chain conformation serves as the structural bridge from primary to higher-order organization in polysaccharides and represents the level most proximate to mechanistic understanding within the SAR paradigm. Polysaccharides are prone to forming helical secondary structures and higher-level assemblies. β-glucan is one of the most intensively studied bioactive polysaccharides. β-(1,3)-glucan is present in plant cell walls, and long-chain forms have the ability to form triple helix structures [134].

**Figure 4:**
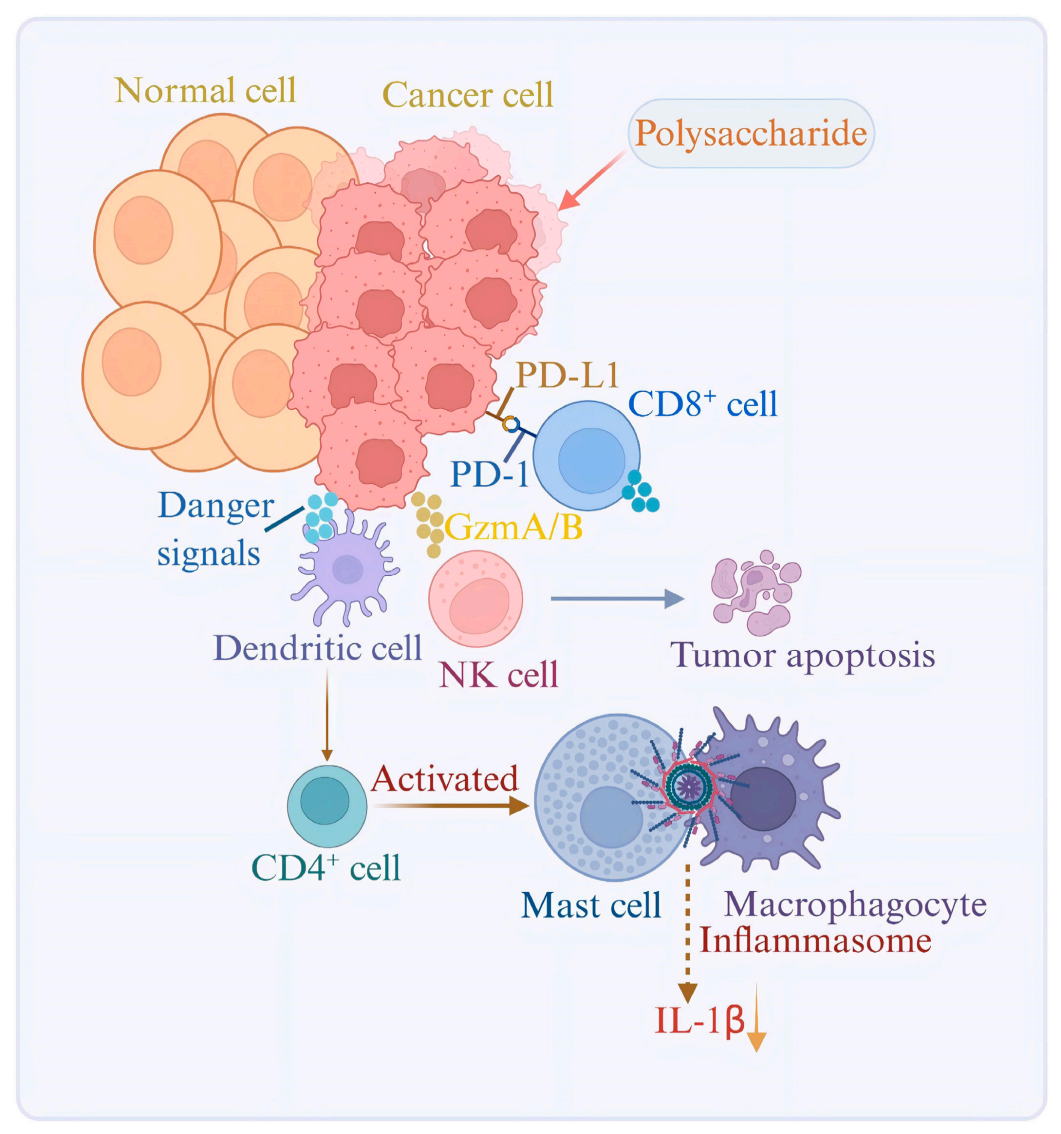
The anti-tumor effect of polysaccharides. This figure demonstrates that polysaccharides have a certain effect on immune cells, thereby influencing the tumor. In the two arrows of IL-1β in the figure, the dotted arrow indicates that the occurrence and development of cancer cells may stimulate the secretion of inflammatory cytokine IL-1β by inflammasomes, while the solid arrow pointing downward suggests that when polysaccharides intervene in cancer cells, it may reduce the secretion of inflammatory factors.

The triple helix conformation can affect the bioactivity of certain polysaccharides. A study compared the structures of three polysaccharides in vinegar-baked *Radix Bupleuri* and their anti-hepatoma activities in cell studies (Figure 4), which suggested that a molecular weight of approximately 16 kDa, a high degree of arabinan polymerization, and a triple helix structure were the key structural features for their activity [100]. Among the four fractions (LPI–IV) isolated from the crude polysaccharides of longan pulp, only LPI possesses both a compact spherical structure and a triple helical structure, while LPII–IV exist in a flexible chain form without a triple helix structure, and LPIII exhibits significantly superior anti-tumor activity to LPI in cell studies, which indicates that the biologically active conformation responsible for anti-tumor and immune-modulating activities in longan polysaccharides may be the flexible chain [105]. This indicates that the more complex structures, such as chain conformation, may play different roles in plant-derived polysaccharides.

Moreover, the conformation of polysaccharide chains possesses a tunable nature. External stimuli, including ultrasound, microwave irradiation, heating, and radiation, are capable of inducing changes in chain conformation [135]. Following subcritical water treatment, *Glycyrrhiza inflata* Batalin polysaccharides transition from a rigid conformation to a flexible one, accompanied by a marked increase in antioxidant activity in chemical assays and immune activity in cell studies [136]. This means that different extraction methods do more than alter chemical composition; they may also modulate physical conformation, thereby either activating or suppressing biological functions.

### 3.7. Higher-Order Structure

Currently, studies on the higher-order structure of polysaccharides, encompassing chain conformation, side chain arrangement, and aggregate morphology, face certain limitations. Scanning electron microscopy (SEM) and atomic force microscopy (AFM) have been used to investigate polysaccharide morphology and aggregation state [137]. Despite observed structure–activity correlations, the path to causal mechanisms remains long.

Accumulating microscopy evidence has shown that bioactive polysaccharides exhibit substantial morphological diversity, with AFM and SEM images revealing structures such as aggregates, fibrous networks, ring-shaped assemblies, and lamellar sheets [138,139,140] (Table 5). For instance, the rod-like stacked aggregates of *Codonopsis pilosula* polysaccharide (CPPS-II) correlate with the strongest anti-tumor and immunomodulatory activities in animal experiments and cell experiments [110]. By observing the morphology of the two polysaccharide components (LBP-1/2) isolated and purified from *Lycium barbarum* fruits, it was found that the semi-networked and fragmented form of LBP-2 exhibited superior antioxidant and tyrosinase inhibitory activities compared to the aggregated, smooth sheet-like and triple helix-structured LBP-1 in chemical assays [99].

Evidently, polysaccharide bioactivity is closely tied to the degree of structural compactness. Only polysaccharides that form a certain degree of polymer structure may exhibit stronger activity; overly compact or overly loose structures seem to show weaker activity. In immunomodulation, systematic evidence is lacking for a mechanism that links higher-order structure (such as the triple helix) to specific receptor binding (such as Dectin-1) and downstream signaling pathway activation [22,141]. It is a well-established area of research that polysaccharides influence host health, including inflammation-related signaling pathways, by modulating the gut microbiota; however, the underlying molecular mechanisms, from higher-order structures to specific functions such as the effects on pathways like NF-κB or JAK-STAT, remain a scientific gap that urgently needs to be explored [142,143].

**Table 5:** Morphological characteristics and biological activity of different components of polysaccharides.

Polysaccharide	Component	Morphological Characteristics	Optimal Component	Bioactivity	Reference
*Codonopsis pilosula* polysaccharides	CPPS-I (<60 wDa)	Complete ring structures and longer chain structures with a few surface pores	CPPS-II	Anti-tumor activity; enhance immune function	[110]
	CPPS-II (60–100 wDa)	Polysaccharide aggregates of varying sizes, rod-like stacked structures, layered structures			
	CPPS-III (>100 wDa)	Aggregates of polysaccharides of varying sizes, with a few pores on their surfaces, form a layered structure.			
Polysaccharides from the Roots of *Isatis indigotica* Fort.	RIP-A1 (1.8 × 10^4^ Da)	Surface roughness with a uniform honeycomb-like polymer particle structure	RIP-A1	anti-inflammatory activity	[116]
	RIP-B1 (7.4 × 10^4^ Da)	Slight surface wrinkling with loosely arranged large-scale layered structures, exhibiting chain-like and irregular spherical conformations.			
	RIP-B2 (9.3 × 10^4^ Da)	The smooth surface exhibits a layered structure with uniformly sized grid-like conformation.			
*Elaeagnus umbellate* Polysaccharides	EUP1 (63 kDa)	Irregular striped structures interspersed with numerous relatively large spherical particles	EUP1	Antioxidant activity, scavenging DPPH, hydroxyl, ABTS and other free radicals	[144]
	EUP2 (38 kDa)	A coarse, flaky structure dotted with numerous small spherical particles.			
*Lycium barbarum fruit* polysaccharides	LBP-1 (115.1 kDa)	Aggregated sheet-like structures, with smooth and compact surfaces, and a triple helix structure	LBP-2	Antioxidant activity	[99]
	LBP-2 (73.5 kDa)	Semi-networked, fragmented form, relatively scattered and fibrous			

Therefore, current research on higher-order structure remains largely at the stage of descriptive observation, lacking in-depth mechanistic analysis at the molecular level. The critical question, how to move from observing aggregate formation to understanding how aggregates bind to receptors, represents an urgent priority for this field.

### 3.8. The Influence of Solution Environment on the Conformation of Polysaccharides

In addition to the aforementioned intrinsic structural parameters (branching ratio, molecular weight, and triple helix integrity), the actual conformation of polysaccharides in aqueous solutions is also highly dependent on solvent conditions, including water molecules and ionic strength [145]. This environmental sensitivity may have direct functional implications. For instance, the triple helix conformation of β-glucan, which is crucial for Dectin-1 receptor recognition, is not an inherent property but is a dynamic state shaped by the surrounding solution environment [145,146,147]. This section further clarifies the influence of physiological and industrial solution conditions on the conformation of polysaccharides by discussing the impact of solvents on their conformation.

Molecular dynamics (MD) simulations provide atomistic details regarding the organization of water molecules around polysaccharide chains and the conformational transitions of polysaccharides in water [148,149,150]. For instance, MD simulations have shown that in aqueous solution systems, more water molecules directly interact with the *Bletilla striata* polysaccharide molecules through hydrogen bonding, and the hydrogen bonds formed within the molecules contribute significantly to maintaining the stability of the polysaccharide structure [151]. Moreover, increasing temperature accelerates the breakage of hydrogen bonds between *Bletilla striata* polysaccharides and water molecules, which to some extent damages their conformational stability [151]. Additionally, in the presence of water molecules, the hydrogen bond count per repeating unit of homogalacturonan pectin is elevated, and it transforms from a double helix structure to a triple helix structure [148]. This suggests that the hydrogen-bonding network between polysaccharides and water molecules may play a crucial role in maintaining the conformational stability of plant-derived polysaccharides. Additionally, changes in environmental conditions (such as solvent composition) have been demonstrated to cause systematic conformational rearrangements in polysaccharide chains by affecting the solvation process [152]. MD simulations of β-glucan conformational behavior in aqueous solution demonstrated that single-stranded β-1,3-glucan with a DP of 24 assembles into aggregates, whereas triple-stranded β-1,3-glucan with a DP of 6 preferentially adopts a double-helical structure [141]. Although longer side chains may trigger local aggregation, the branching introduced by β-1,6 glycosidic linkages confers flexibility and strengthens hydrogen bond interactions with water [141].

Additionally, the ionic strength of the surrounding solution also exerts a critical influence on modulating polysaccharide conformation [153]. For non-ionic polysaccharides, such as guar gum, earlier studies have shown that their intrinsic viscosity hardly changes with the ionic strength, which reflects the lack of charged groups on the main chain [154]. One study used rheological measurements and atomic force microscopy to confirm that Mg^2+^, K^+^ and Na^+^ can all induce the formation of gels from soy hull soluble polysaccharide, but the inducing effects of different ions are significantly different, and the network structure formed by Mg^2+^ is more stable and uniform than that formed by K^+^ and Na^+^ [155]. Moreover, the SHSP60 component of soy hull soluble polysaccharide forms aggregates as the concentration of metal ions increases and no longer forms a network structure, and even at 0.40 mol/L, no gel can be formed [155]. It is indicated that the conformational changes of plant-derived polysaccharides are not only related to ionic strength, but also associated with the type of counterions. Temperature also influences these effects, for example, a study has shown that although the *Descurainia sophia* seed gum possesses high viscosity at relatively low concentration levels, its characteristic viscosity remains basically unchanged within a very wide range of ionic concentrations, and with increasing solution temperature, both the characteristic viscosity and the molecular chain size (curling size) of the silymarin gum decrease [156].

In conclusion, the conformation of polysaccharides may have a certain specific balance among water molecules, ionic strength and temperature conditions [157,158]. Therefore, when studying the biological activity of polysaccharides, the solution conditions during the formation of the conformational state need to be carefully considered. If the research only reports the bioactivity of polysaccharides without indicating the solvent environment, it may overlook the key factors that affect the correlation between structure and bioactivity. We suggest that future research should at least clearly specify the solvent system, ionic strength, pH value and temperature used for conformational and biological activity assessment.

### 3.9. Contradictory Findings and Methodological Limitations in SAR Studies

Despite the general trends described in the preceding sections, the relationship between structural parameters and bioactivity is not always consistent across studies. For molecular weight, some studies report that low-molecular-weight fractions exhibit stronger immunomodulatory and antioxidant activities [107,109], whereas others show that medium- or high-molecular-weight fractions are more active [110,112]. For chain conformation, triple-helix structures have been reported as essential for anti-tumor activity in some polysaccharides [100], while flexible chains appear more important in others [105]. These contradictions suggest that structural parameters do not act independently but rather interact with each other when influencing bioactivity. For example, the effect of molecular weight cannot be interpreted in isolation from chain conformation, and the degree of branching is influenced by the type and distribution of the branches, which in turn affects the conformation and solubility of the molecular chain [129]. Uronic acid content contributes to negative charge density, which affects metal ion chelation and receptor binding, but this effect is also influenced by the position of uronic acid residues within the backbone [128]. Therefore, no single structural parameter can be sufficient to predict bioactivity across all polysaccharides, and multi-factor analysis combining structural characterization, computational modeling, and functional assays may be needed to establish reliable SAR. Based on these interactions, we hypothesize that (1) the optimal molecular weight for a given bioactivity may be influenced not by molecular weight alone, but by the balance between chain flexibility and ordered domain formation; (2) the effect of monosaccharide composition may be context-dependent; the same monosaccharide can act as either a recognition motif or a steric hindrance depending on its position; and (3) an optimal branching degree may exist, reflecting a trade-off between solubility and receptor binding affinity. These hypotheses warrant systematic experimental validation.

### 3.10. Characterization Methods and Their Limitations

These methodological challenges are also related to the limitations of current characterization techniques. The structural characterization of plant-derived polysaccharides relies on a combination of chemical, chromatographic, spectroscopic, and microscopic methods. Commonly used techniques include size-exclusion chromatography (SEC) and high-performance liquid chromatography (HPLC) for molecular weight determination and monosaccharide composition analysis, gas chromatography-mass spectrometry (GC-MS) for methylation analysis of glycosidic linkages, Fourier-transform infrared spectroscopy (FT-IR) for functional group identification, nuclear magnetic resonance (NMR) for glycosidic linkage and anomeric configuration analysis, and scanning electron microscopy (SEM) and atomic force microscopy (AFM) for morphological observation [106,137,159].

However, each method has inherent limitations. SEC suffers from calibration-standard mismatch (sample vs. standard hydrodynamic volume) and non-size-exclusion effects, both of which distort molecular weight determination [160]. HPLC-based monosaccharide analysis requires time-consuming derivatization, may struggle to detect certain monosaccharides such as ketoses and acidic sugars, and can suffer from interference caused by residual derivatization reagents [161,162]. GC-MS requires derivatization and may not distinguish anomeric configurations without additional NMR data [163,164]. FT-IR provides only qualitative functional group information and cannot resolve fine structural details [165]. NMR requires relatively large sample amounts and high purity, and complex polysaccharides with heterogeneous linkages often produce overlapping signals that are difficult to assign [164]. SEM and AFM observation of polysaccharides generally requires drying, yet the resulting dried or surface-state images may not accurately represent their true conformation and conformational change process in solution [166,167]. Moreover, none of these methods alone can fully resolve higher-order structures such as triple-helix conformations or aggregate morphology in solution.

To address these limitations, advanced analytical techniques are increasingly being applied to polysaccharide characterization. Circular dichroism (CD) spectroscopy can provide information on chain conformation and helical structure in solution [168,169]. Advanced NMR techniques, including two-dimensional and solid-state NMR, can resolve overlapping signals and provide more detailed information on glycosidic linkages and anomeric configurations [170]. Small-angle X-ray scattering (SAXS) can resolve the overall shape, size, and aggregation state of polysaccharides in solution, complementing the surface-only information provided by SEM and AFM [171]. However, no single method can fully resolve polysaccharide higher-order structures; integrating multiple complementary techniques is essential for reliable conformational analysis.

These methodological limitations partly explain why cross-study comparisons of polysaccharide structure–activity relationships remain difficult and why the mechanistic link between higher-order structure and bioactivity is still poorly understood. Therefore, standardizing the reporting of characterization methods and experimental conditions may be of crucial significance for achieving reliable comparisons among multiple studies.

## 4. Structural Modification

**Figure 5:**
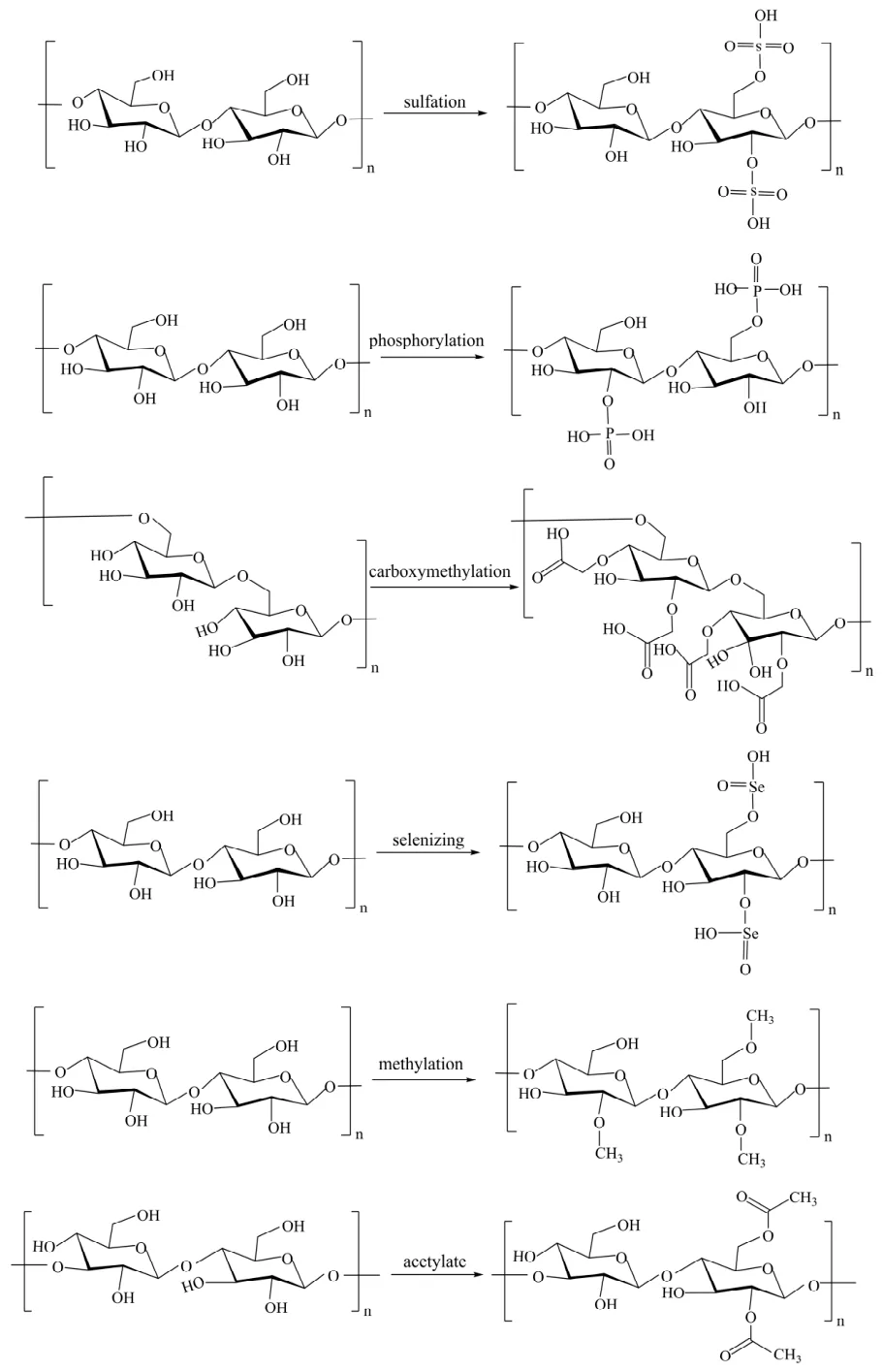
Polysaccharide structure modification methods. It can be clearly observed that polysaccharide modification occurs at specific groups or sites, suggesting that more modification sites need to be further discovered.

Polysaccharides of plant origin frequently have weak bioactivity due to high molecular weight, low branching, limited solubility, or missing functional groups. Structural modification aims not just to enhance activity but to actively validate SAR principles. By chemically modifying SAR-derived key parameters, known activities can be amplified and new functions activated. Thus, structural modification bridges structural analysis and functional application, which may represent the essential step from descriptive science to design science. Current polysaccharide modification methods include sulfation, phosphorylation, carboxymethylation, selenation, methylation, acetylation, and metal ion complexation (Figure 5). These introduce different functional groups (-SO_3_H, -Se, -CH_3_, -COCH_3_) or chelate metal ions, thereby changing molecular weight, charge density, hydrophilic/hydrophobic balance, and chain conformation, and thus tuning bioactivity [172] (Table 6).

### 4.1. Sulfation: The Impact of Negative Charge Density on Bioactivity

Sulfation represents a chemical modification strategy wherein the hydroxyl groups of polysaccharides are substituted with sulfate groups (-SO_3_H). The most commonly employed procedures comprise the method using chlorosulfonic acid and pyridine, the concentrated sulfuric acid method, and the method employing sulfur trioxide and pyridine [195]. Through sulfation, a certain correlation may be observed between negative charge density and specific biological activity.

**Figure 6:**
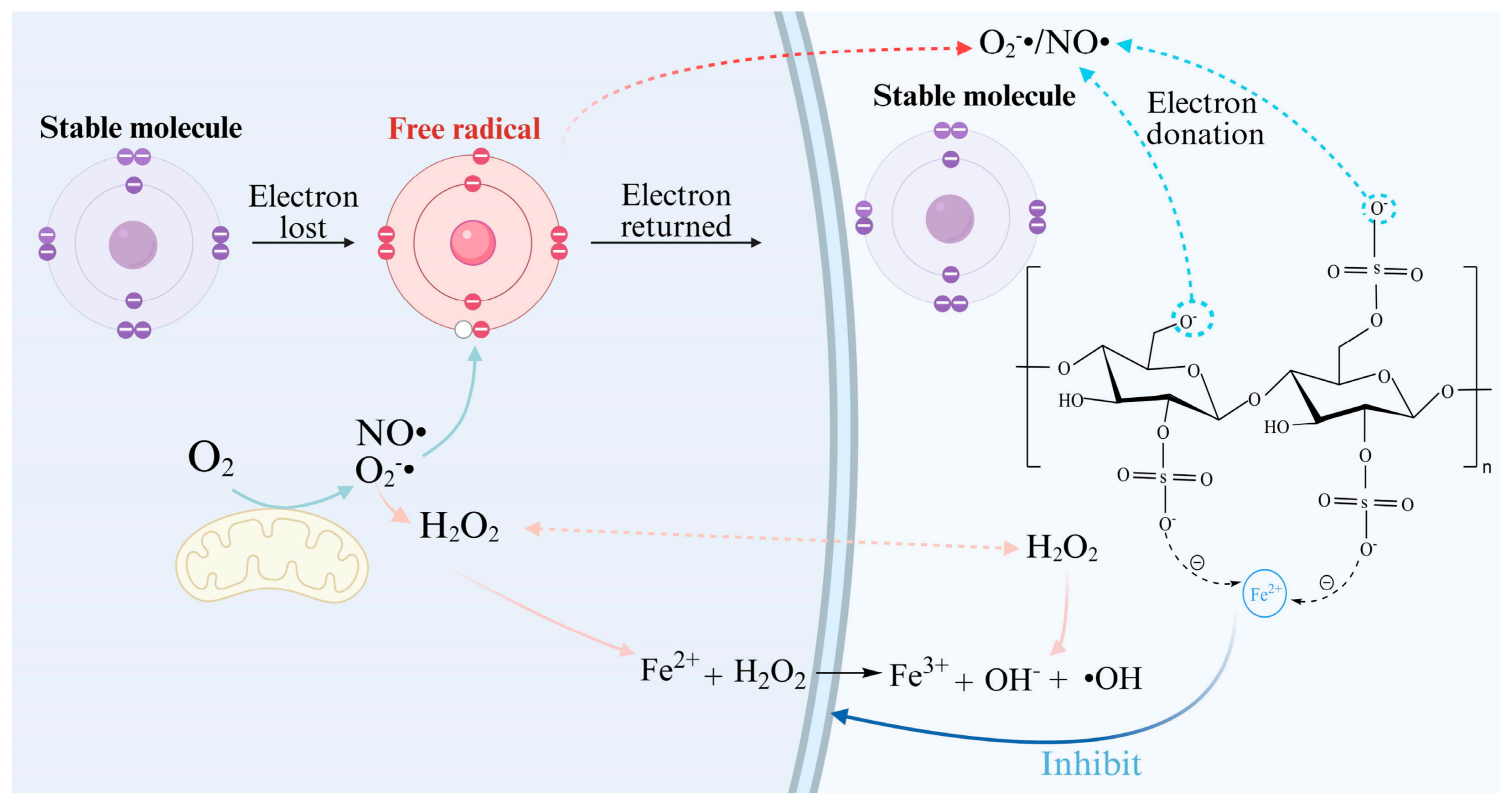
The mechanism of polysaccharide sulfate’s antioxidant effect. This figure suggests that the sulfation process enhances the activity of polysaccharides possibly due to the increase in polarity and metal chelation.

Sulfation serves as an effective means of modulating polysaccharide bioactivity. It has been demonstrated that glucuronic acid residues in polysaccharides may facilitate metal ion chelation and confer strong free radical scavenging capacity [173]. In the case of dandelion root polysaccharides, which contain glucuronic acid, sulfation at the C6 hydroxyl position not only markedly boosts their antioxidant and hypoglycemic effects in chemical assays but also promotes the proliferation of beneficial intestinal bacteria [173]. The presence of uronic acids may facilitate metal ion chelation, as the carboxyl groups (-COO^−^) possess a high charge density capable of coordinating with divalent metal ions including Fe^2+^ and Cu^2+^, thereby potentially suppressing their catalytic role in free radical generation [196]. Similarly, the potent antioxidant capacity of sulfated polysaccharides is likely attributable to the chelation of metal ions by their negatively charged, high-density sulfate groups (-OSO_3_^−^) [197]. In chemical assays, sulfated blackcurrant polysaccharide derivatives not only exhibit excellent free radical scavenging properties, but also significantly enhance their iron ion chelating capacity, and this capacity is positively correlated with the sulfate group substitution degree [198]. Another study found that after removing endogenous metal ions from natural sulfated polysaccharides through EDTA treatment, the chelating activity of Fe^2+^ actually increased, suggesting that the sulfate groups themselves have the ability to bind metal ions [199]. (Figure 6). Naturally occurring sulfated polysaccharides from *Sagittaria trifolia* (PST) possess inherent antioxidant and procoagulant activities [174]. In chemical assays, following sulfation, its antioxidant capacity exhibits a biphasic trend. It initially increases and then decreases, with moderate substitution degrees yielding the optimal effect [174]. More strikingly, sulfation converts PST’s native procoagulant activity into anticoagulant activity. This transformation bestows an entirely new pharmacological function [174]. These observations imply that the degree of modification, which is the substitution degree, acts as a major contributor to both the type and intensity of biological activity. An optimal range exists for this parameter. In other words, a higher substitution degree does not necessarily translate into better activity.

Furthermore, polysaccharide activity is also associated with the content of sulfate groups (-OSO_3_^−^). The degree of substitution of the sulfated purple glutinous rice bran (*Oryza sativa* L.) polysaccharides ranged from 0.01 to 0.56, and the samples with the highest degree of substitution (0.53 and 0.56) displayed the strongest stimulatory activity against RAW 264.7 macrophages, demonstrating that sulfation significantly enhanced the immunomodulatory activity of the polysaccharides [200]. Notably, the antioxidant activity of most of the sulfated products of this polysaccharide is weaker than that of the crude polysaccharide, which is speculated to be due to the degradation of the anthocyanins that are connected to the polysaccharides during the sulfation process, indicating that the antioxidant activity of the polysaccharide is not only influenced by its own structure but may also be related to the anthocyanins to which it is connected [200]. Sulfation also enhanced the antioxidant effect of *Zizania latifolia* polysaccharides, and this effect was proportional to the substitution degree, among which the polysaccharide with a substitution degree of 15.1 ± 2.50 exhibited strong hydroxyl and DPPH free radical scavenging capabilities in chemical assays [201]. It is demonstrated that the introduction of sulfate groups (especially when reaching the optimal substitution degree) can effectively enhance the antioxidant and immunomodulatory activity of plant-derived polysaccharides. Moreover, within a certain degree of substitution range, the enhancement of activity may correlate positively with the sulfate group content/degree.

Therefore, sulfation modification demonstrates that sulfate density, negative charge density, and metal ion chelation ability are intrinsically linked to antioxidant, hypoglycemic, and anticoagulant activities.

### 4.2. Phosphorylation: Bidirectional Optimization of Chain Conformation and Water Solubility

Phosphorylation adds phosphate groups (-PO_3_H_2_) using reagents like phosphorus oxychloride, phosphate salts, and phosphorus pentoxide [202]. This modification not only alters the chemical composition of the polysaccharide chain, but may also induce changes in the conformation of the polysaccharide chain. The polysaccharide from *Asarum sieboldii* Miq (ASP), which does not exhibit a triple helix conformation in its natural form, acquires this structural feature upon phosphorylation, a modification that is simultaneously accompanied by a significant enhancement in its scavenging activities against both DPPH and ABTS radicals in chemical assays [178]. Phosphorylation can alter the charge distribution and steric hindrance of polysaccharide chains. Wang et al. documented that the introduction of -PO_3_H_2_ groups substantially enhances chain stiffness through electrostatic effects, which in turn modifies its conformation in solution [203]. Hansen et al. further demonstrated through molecular dynamics and NMR studies that phosphorylation exerts a steric effect that restricts the conformational space of glycosidic linkages [204]. Phosphorylation of psyllium seed polysaccharide increased its swelling index by 90.26% and imparted adhesion performance comparable to carbomer, highlighting its favorable physicochemical properties and promise as a new excipient [205]. Phosphorylated *Astragalus* polysaccharide exhibits anti-dog hepatitis A virus activity, which increases the survival rate to 33.5 percent in cell studies [177].

Consequently, phosphorylation offers a way to activate polysaccharides that have low activity resulting from a lack of ordered conformation. The activation is achieved through the induction of helical structures. However, traditional phosphorylation is limited by its reliance on toxic solvents and harsh reaction conditions, factors that make it less conducive to food and pharmaceutical applications [206]. Looking forward, the exploration of mild and site-selective enzymatic phosphorylation methods represents a promising alternative, as such approaches may enable more precise structural modulation [206].

### 4.3. Carboxymethylation: An Important Method for Improving the Water Solubility of Polysaccharides

Carboxymethylation adds carboxymethyl (-CH_2_COOH) groups to polysaccharides using chloroacetic acid and sodium hydroxide [202]. This modification improves aqueous solubility, a condition essential for polysaccharide activity.

**Figure 7:**
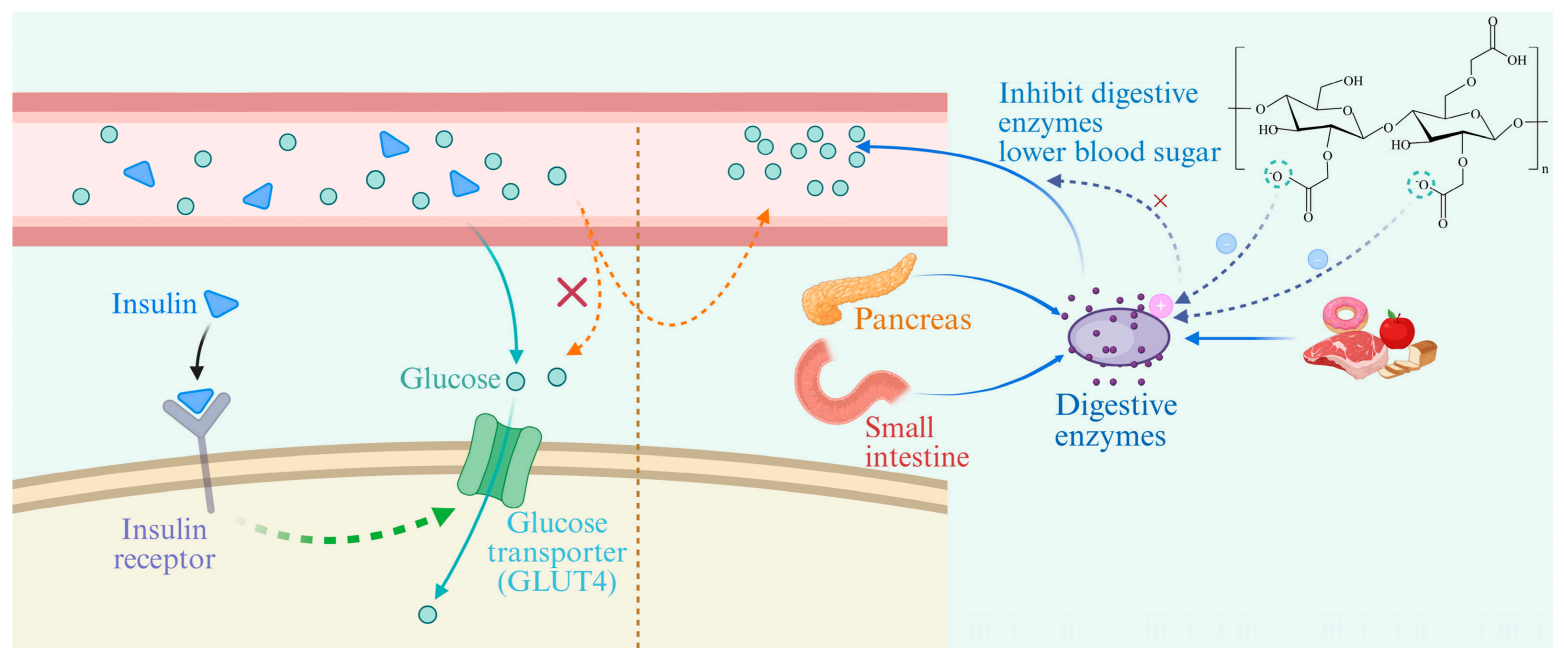
Carboxymethylated polysaccharide hypoglycemic effect. This figure suggests that the hypoglycemic effect of carboxymethylation may be related to the increase in polarity and the presence of charge. The "×" path in the figure indicates that in abnormal situations, the monosaccharides produced during food digestion and hydrolysis cannot be effectively absorbed by cells, and thus remain in the bloodstream.

For carboxymethylated gel polysaccharide, the degree of substitution (DS) below 0.20 retains gelling ability, whereas the DS above 0.36 significantly increases water solubility and forms a stable transparent solution [207]. Several lines of evidence support the effectiveness of carboxymethylation. The surface of carboxymethylated corn bran polysaccharide particles was loose and the structure was changed; in chemical assays, the modified polysaccharide displayed remarkable antioxidant properties, and in cell studies, it exhibited potent tumor-inhibitory effects [181]. Note that the corn bran polysaccharide extract also contains phenolic substances; within a certain degree of substitution, increasing sodium hydroxide concentration raises the substitution degree while lowering the total phenolic content, and as phenolics decrease, DPPH scavenging and ferric ion chelating activities increase, indicating that the biological activity may arise from carboxymethyl substitution rather than the phenolic compounds themselves, and suggesting that rice bran polysaccharide activity may not be affected by certain impurities [181]. Guar gum from *Cyamopsis tetragonoloba*, after carboxymethylation at DS = 0.64, showed significantly higher DPPH scavenging activity in chemical assays and antibacterial activities in the microbiological assays than the unmodified polymer [182]. Carboxymethylation of *Chaenomeles sinensis* polysaccharides changed their surface from a smooth morphology to a sheet-shaped porous structure, and the modified polysaccharide showed significantly enhanced anti-inflammatory and antioxidant responses that effectively treated ulcerative colitis in animal studies [184]. When the polysaccharides of *Sarcandra glabra* (Thunb.) Nakai were subjected to carboxymethylation treatment, the water solubility of the insoluble crude polysaccharides was improved, and the IC_50_value for α-glucosidase reached 146.06 μg/mL, which was stronger than the positive control drug acarbose (317.45 μg/mL) in chemical assays [208](Figure 7). These observations collectively indicate that carboxymethylation of polysaccharides can both alter their morphology and enhance their functional activities.

Carboxymethylation modification confirms the existence of an intrinsic relationship among water solubility, bioavailability, and biological activity. For natural polysaccharides that are characterized by poor water solubility, carboxymethylation might be the preferred modification method, since solubility is a prerequisite for their biological activity to be exerted.

### 4.4. Seleniumization: The Synergistic Enhancement of Trace Elements and Polysaccharides

Selenopolymer is an organic selenium compound in which selenium elements are introduced into the polymer chain. This compound, which possesses the dual activities of selenium and polysaccharides, may have superior biological activity and be more easily absorbed by the human body [209]. Common methods for the preparation of selenopolysaccharides include the sodium selenite nitric acid method and the acetic acid selenium dioxide method. In terms of selenium incorporation, selenium may be bound to the polysaccharide chain via β-1,3 or α-1,4 glycosidic linkages, or it may replace the pyran ring oxygen atom [202,210].

**Figure 8:**
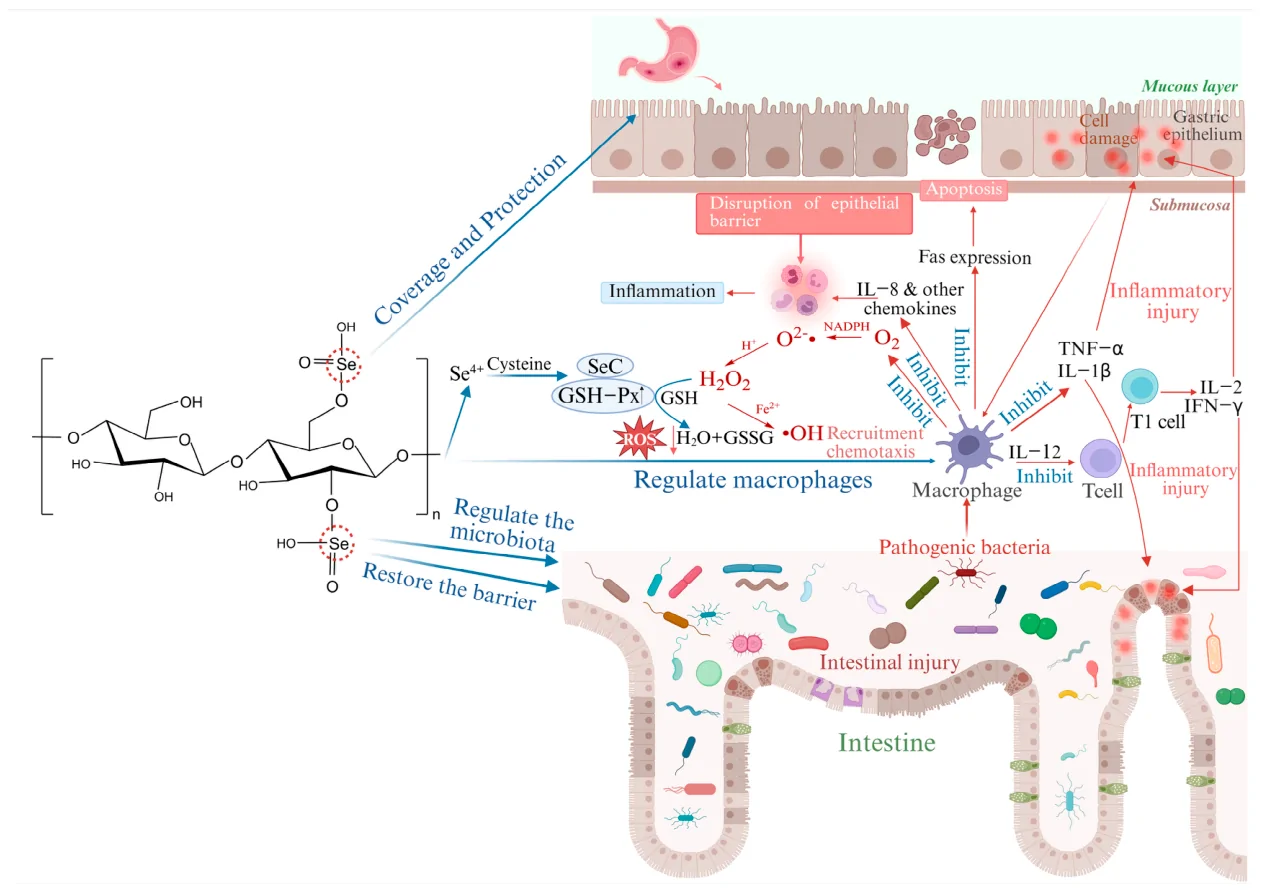
The protective effect of selenated polysaccharides on the gastrointestinal tract. The figure indicates that polysaccharide selenization not only enhances the biological activity of polysaccharides such as anti-inflammatory effects, effects on the microbiota, and protection against damage through the release of selenium, but also has other beneficial effects.

Following selenization treatment, the particle size of the modified Astragalus polysaccharides (APS) decreases, and its surface morphology transforms into a randomly distributed porous structure [159]. In addition to these physical changes, the selenated APS exhibits enhanced antioxidant activity and provides protective effects on human renal proximal tubular epithelial cells (HK-2) [159]. Similarly, after *Codonopsis pilosula polysaccharides* are processed with selenium, the incorporated selenium exists in two forms, namely O-Se-O and O-H…Se, and during simulated gastric digestion, the selenated polysaccharide exhibits a strong ability to release selenium, in addition to which it can repair gastric mucosal damage [179] (Figure 8). The particle size and thermal stability of selenium-modified ordinary tea polysaccharides were reduced, the derivatives showed altered appearance but retained their main functional groups and secondary structure, with improved antioxidant and hypoglycemic activities in chemical assays [180]. Furthermore, functionalization of selenium nanoparticles with *Sonneratia caseolaris* (L.) leaves polysaccharide not only enhanced their stability but also significantly improved their antioxidant activity in chemical assays and antibacterial activity in the microbiological assays [211].

Selenization provides evidence for the mechanism of synergistic effects of trace elements, dual biological activities, and enhanced bioavailability. For polysaccharides that need to enhance their antioxidant, immunomodulation, and anti-aging effects, selenium modification can be regarded as a good approach.

### 4.5. Methylation: Tuning of Hydrophobicity and Interfacial Activity

Methylation converts hydroxyl groups (-OH) into methoxy groups (-OCH_3_), and it is not only a critical tool for the structural analysis of polysaccharides (used to decipher the connection mode of glycosidic linkages), but also a chemical modification method [202,212]. Methylation can also affect hydrophobicity.

After soy hull polysaccharide undergoes methylation, its surface hydrophobicity increases, a modification that results in a decrease in the amount of polysaccharide in the aqueous phase as the degree of methylation increases [185]. What this decrease achieves is the facilitation of SHP migration to the oil phase, which is a critical step that enhances the emulsifying ability of the polysaccharide [185]. Xu et al. produced methylated *okra* polysaccharides via methanol/hydrochloric acid esterification, increasing the degree of methyl-esterification (DM) from 12.94% to 41.50%, which reduced molecular weight and zeta potential but enhanced hydrophobicity and interfacial tension-reducing ability, while its emulsifying capacity was optimal only under appropriate conditions (such as molecular weight, chain conformation, monosaccharide ratio, and DM) [213].

Methylation modification mainly demonstrates that there is an interrelationship among the hydrophobic hydrophilic balance, surface activity, and emulsifying ability of polysaccharides. What this finding implies is that for polysaccharides that need to be used as emulsifiers or delivery carriers, methylation represents an effective optimization strategy. However, the chemical methods of introducing new groups through methylation to alter the activity of polysaccharides have been less studied. The main approach is to use methylation methods for structural analysis of polysaccharides, while the chemical modification requires further research.

### 4.6. Acetylation: Effects on Amphiphilicity and Immune Activity

The acetyl group (-COCH_3_) is a hydrophobic moiety that imparts amphiphilicity to hydroxyl-rich polysaccharides. Variations in acetylation sites and the degree of substitution may alter the chain conformation and spatial architecture of polysaccharides, thereby leading to differences in water solubility and bioactivity, and the commonly used reagent for acetylation is acetic anhydride [214].

In chemical assays, acetylated litchi pericarp polysaccharides enhanced the scavenging activities against hydroxyl free radicals and superoxide anion radicals, with the hydroxyl radical scavenging activity being stronger than both its reducing power and superoxide anion scavenging activity at the same polysaccharide concentration [215]. Acetylation significantly alters the surface topography and dissolvability of *Dendrobium officinale* polysaccharides, and the resulting derivatives facilitate immunoglobulin secretion, increase immune cell populations, and thereby enhance overall immunity [187]. Acetylated polysaccharides can not only improve antioxidant and immunomodulatory activities, but also decrease blood glucose levels and exert a preventive effect against diabetes [189]. The more porous surface structure of acetylated polysaccharide from *Houttuynia cordata* rhizome increased the solubility by 1.83 times, and the enhanced inhibition of α-glucosidase correlated with superior glycemic control [189]. Following acetylation, *Rhododendron dauricum* leaves polysaccharides exhibited decreased molecular weight, loss of triple-helix conformation, and increased chain ductility, which were correlated with improved anti-complementary activity, as reflected in enhanced complement inhibition in cell studies [188]. Acetylation provided further confirmation of the close correlation that exists between conformational changes and changes in biological activity. In addition, this modification revealed the interaction rules that shape the relationships among amphiphilicity, solubility, bioavailability, and pharmacological activity. Therefore, for polysaccharides that need to enhance their biological activity by improving their solubility, acetylation represents an effective strategy.

### 4.7. Metal Ion Complexation: Structural Form and Synergistic Delivery of Trace Elements

The complexation of polysaccharides with metal ions including Fe, Zn, Mg, Cr, and Pt may alter their structure and enhance their bioactivities, including antioxidant, antibacterial, anti-tumor effects, and improvement of blood sugar and lipid levels, while simultaneously supplementing the body with deficient trace elements [216,217]. Essential trace elements such as Fe, Cu, and Zn are critical for sustaining various physiological functions in the human body, regulating the metabolic balance, enhancing immunity and preventing diseases. Arabic gum-stabilized radioactive gold nanoparticles form a polysaccharide-coated colloid that exhibits good stability and low cytotoxicity, while also accumulating at tumor sites and inhibiting tumor growth in vivo, holding potential for applications in tumor diagnosis and brachytherapy [218]. In another study, crude polysaccharides from crocodile flower were used as a green reducing agent and stabilizer to synthesize silver nanoparticles, which not only exhibited excellent stability in solution but also showed significant anti-proliferative activity against MCF-7 breast cancer cells [219].

Arabic gum was used as a polysaccharide stabilizer to prepare Cu nanodots/Arabic gum hybrids, and the composite material could modulate apoptosis-related proteins, significantly reducing the survival rates of A549 and HEK239T cells [192]. The Qingzhuan Dark Tea polysaccharide–cobalt complex not only exhibits higher thermal stability but also enhances the scavenging capabilities against DPPH, hydroxyl, and ABTS free radicals in chemical assays; furthermore, after cobalt addition, most of the nucleic acids or conjugated proteins of this polysaccharide–cobalt complex were removed [220]. Moreover, cobalt is a trace element known to promote bone growth, and this polysaccharide–cobalt complex shows the most significant effect on MC3T3-E1 cell proliferation when the cobalt concentration is 2 ppm, and may therefore have potential application value in promoting bone regeneration [220]. When soluble soybean polysaccharides are chelated with zinc, they transform from a filamentous structure to a cluster-like structure with cracked fragments on the surface, while the triple helix conformation changes to an irregular random coil conformation, accompanied by an increase in thermal stability [221]. Moreover, through an in vitro simulation of gastrointestinal digestion, the release rate of Zn^2+^ reached 68.87% after 2 h of digestion [221]. This indicates that the chelation of polysaccharides with zinc may not only alter the structure but also provide a basis for the research and application of novel zinc supplements. Under the microscope, ginger peel polysaccharides (GPs) appeared as irregular, rough, porous fragments [194]. After zinc derivatization, the GP-Zn^2+^ complex assumed a compact appearance with a relatively flat sheet structure, while its crystallinity and asymmetry decreased, though thermal stability remained largely unchanged [194].

Therefore, metal ion complexation reflects the interrelated changes in molecular morphology, structure, physicochemical properties, and biological activity. This modification method may enhance the activity of polysaccharides while supplying trace elements, making it a notable modification strategy.

### 4.8. Integration of Structural Modification Strategies

Structural modification is not a blind chemical operation, but a rational design based on the rules of SAR. Each modification method may affect the biological activity by altering specific structural parameters. Clarifying the correlation patterns between modification methods, molecular structures and biological activities may represent an important step in the shift from trial-and-error exploration to predictive verification in research. In light of the above findings, we preliminarily formulated the selection strategies for polysaccharide structural modification (Table 7).

### 4.9. Stability of Modified Polysaccharides Under Physiological Conditions

In order to transition the modified polysaccharides from laboratory research to clinical or nutritional health product applications, their stability under physiological conditions, especially in the gastrointestinal environment, is a key factor affecting their efficacy [222]. When administered orally, polysaccharides are exposed to gastric acid, digestive enzymes (gastric pepsin, intestinal trypsin and α-amylase) and bile salts, all of which may cause degradation or structural changes to the polysaccharides before they reach the target site [223,224]. Recently, researchers have turned their attention to the oral absorption mechanism and stability of polysaccharides, and have attempted to protect their activity in the gastrointestinal tract through strategies such as nano-delivery systems [225].

#### 4.9.1. Gastric Acid Stability

Most oral polysaccharides, which are susceptible to gastric acid degradation and mainly act in the intestine, can be modified with specific chemical moieties (such as carboxymethyl and mercapto groups) to enhance gastric acid stability, thereby maintaining structural integrity during gastric transit and enabling subsequent intestinal-targeted release or functional expression [226]. A polysaccharide drug delivery system that prevents the release of drugs in the upper digestive tract while being easily degraded by colonic bacterial enzymes has been reported [227]. Studies have shown that the optimized polysaccharide precursor drugs exhibit extremely low release rates (less than 4%) in both strong acid (pH 1.2) and weak acid (pH 6.8) environments, suggesting that they may have good stability in the gastrointestinal tract environment [228]. Carboxymethylation confers pH responsiveness to polysaccharides such as guar gum, enabling them to suppress drug release and maintain stability in gastric acid while swelling or degrading at intestinal pH (5.0–7.4) for targeted release [229,230]. The carboxymethylated neutral tamarind seed polysaccharides were endowed with anionic properties and pH responsiveness, and were then prepared into stable oral delivery nanoparticles through the ionic gelation method [231]. These nanoparticles displayed pH-dependent swelling behavior, remained stable in simulated gastric fluid, and achieved sustained drug release for up to 48 h in the intestinal environment, demonstrating good biocompatibility [231]. These properties have been employed to develop pH-sensitive delivery systems for transporting bioactive compounds that are easily degraded in the stomach [232].

#### 4.9.2. Enzymatic Stability

The sensitivity of modified polysaccharides to enzymatic degradation is closely related to the modification type and the degree of substitution [233,234]. The tamarind seed polysaccharides (with molecular weights of 176.68 kDa and 34.34 kDa) modified by enzymatic hydrolysis (which remain undegraded in gastric and intestinal digestive juices) accelerate their degradation when entering the colonic fermentation stage, and the degradation rate increases as the degree of modification decreases [235]. This indicates that the different molecular weight tamarind seed polysaccharides obtained through enzymatic hydrolysis affect the degradation patterns of polysaccharides mediated by gut microbiota. It has been reported that starch polysaccharides possessing a substitution degree of 0.9–2.0 for acid anhydride and 0.2–0.8 for octanoic acid exhibit resistance to enzymatic hydrolysis by α-amylase under pH 5.5 conditions, while naturally unmodified starch undergoes rapid degradation [236]. It is suggested that the type of modification and substitution degree exert a marked influence on the stability of polysaccharides in the digestive tract enzyme environment.

Furthermore, the incorporation of charged groups (including sulfate and phosphorylation groups) may affect enzymatic hydrolysis by influencing the electrostatic repulsion between the polysaccharide and the enzyme. For example, a study on the polysaccharides of hydroxylated tamarind (*Tamarindus indica* L.) seeds revealed that the zeta potential of the polysaccharides significantly decreased from approximately −0.70 mV to −43.0 mV after being hydroxylated, and the enzymatic degradation activity was markedly reduced, indicating that the charged groups hindered the enzymatic hydrolysis process [237]. Furthermore, a study on the hydrolysis of sugarcane bagasse lignocellulosic material revealed that under a pH of 5.5, the negative charge density on the substrate and the cellulase surface increased, enhancing the electrostatic repulsion between them, which reduced ineffective adsorption, that is, decreased the non-productive adsorption of cellulase on lignin, thereby boosting the overall enzymatic hydrolysis efficiency, and as a result, the enzymatic hydrolysis efficiency of sugarcane bagasse increased from 56.7% to 65.5% [238]. On the other hand, charged groups may also affect enzyme–polysaccharide interactions by altering their conformation, such as the change in water solubility and spatial structure of *Cyclocarya paliurus* leaf polysaccharide, enabling it to more effectively bind and inhibit the activity of α-glucosidase [239].

Therefore, the influence of modification on the sensitivity to enzyme hydrolysis is intimately linked to the type of modification, degree of substitution, the nature of the digestive enzyme, and the resulting complex electrostatic interactions [240,241].

#### 4.9.3. Summary of Physiological Condition Stability

In conclusion, the stability of the gastrointestinal tract exerts a critical influence on the fate of polysaccharides. The design of polysaccharide efficacy studies needs to take into account the harsh environment within the gastrointestinal tract and confirm their stability in physiological fluids, as structural changes may affect pharmacokinetics and immunogenicity [242]. We suggest that future research should at least include in vitro simulated gastrointestinal digestion tests to assess the stability of modified polysaccharides under physiologically relevant conditions.

### 4.10. Toxicology and Biocompatibility of Modified Polysaccharides

The chemical modification of polysaccharides typically involves the use of various reaction reagents (chlorosulfonic acid, pyridine, sulfur trioxide, chloroacetic acid, sodium hydroxide and phosphorus pentachloride), some of which are toxic, corrosive or potentially carcinogenic [195]. Completely removing these reagents and their by-products from the final-modified polysaccharides is technically challenging and is a key safety consideration in any clinical or food application.

#### 4.10.1. Toxicity of Residual Reagents

The commonly employed sulfating reagents, including chlorosulfonic acid and sulfur trioxide, are highly corrosive and generate acidic by-products [195,243]. Pyridine is often used as a catalyst and acid remover for sulfonation and phosphating, but it is known to be a neurotoxin and a hepatotoxic substance [21,244]. Furthermore, the use of chemical reagents may lead to environmental pollution issues [245]. Therefore, even if polysaccharide chemical modification has excellent effects, the toxicity of the chemical reagents, environmental pollution, and toxicity assessment all need to be taken into consideration.

The regulatory frameworks such as ICH Q3C classify these reagents and solvents based on the allowable daily exposure limits for residual solvents, ensuring that drugs do not contain residual solvents above the levels supported by safety data, and this principle also applies to pharmaceutical-grade modified polysaccharides, which require strict quality control [246]. Despite these requirements, few published studies report residual reagent levels, making it difficult to assess the safety profile of the final product. Therefore, strengthening the management and detection of the toxicity and safety of modified polysaccharides is very important. At the same time, in the agricultural, industrial, and biomedical fields, especially in the food industry, comprehensive toxicity testing procedures must be established when using modified polysaccharides [247].

#### 4.10.2. Biocompatibility

Chemical modification endows polysaccharides with new functional properties, while also revealing their advantages and disadvantages in terms of biocompatibility. This establishes a solid foundation for the application of modified polysaccharides in the biomedical field. Studies have shown that certain natural plant-derived polysaccharides inherently possess excellent biocompatibility. Plant-derived polysaccharides are highly valued due to their properties such as biocompatibility, reduced toxicity, and excellent biocompatibility [248]; natural polysaccharide hydrogels are also extensively employed in biomedical applications owing to their good biocompatibility and physical properties similar to those of extracellular matrix [249].

The influence of chemical modification on biocompatibility is not a single-directional effect but rather presents a complex duality. On one hand, chemical modification has been shown to sustain or even augment the biocompatibility of polysaccharides. For example, after conducting high iodic acid oxidation modification on tragacanth gum, the modified polysaccharide still exhibited an extremely low hemolysis rate and excellent cell safety, without causing any significant cytotoxicity [250]. Chemical cross-linking, as a common method to enhance the mechanical strength of biological polymer scaffolds, has better cell adhesion and growth than biocompatibility compared to cross-linking agents [251]. The natural polysaccharides themselves possess excellent biocompatibility, renewability, low immunogenicity, and degradability, making them excellent materials for drug delivery [252].

On the other hand, chemical modifications may also have a negative impact on biocompatibility. For instance, natural *Astragalus* polysaccharides (containing 18.0% sulfate groups) can effectively activate RAW264.7 macrophages and NK cells; however, after desulfation (with the sulfate groups reduced to 8.1%), the activation level of NK cells significantly decreases, indicating that by removing the sulfate groups, the potential excessive immune activation risk of natural polysaccharides can be reduced, thereby enhancing their biocompatibility [253]. Additionally, sulfated Astragalus polysaccharides have high anti-HIV activity and lower cytotoxicity [254]. When certain chemical modification groups are introduced into polysaccharides, there may be certain risks of cytotoxicity.

In conclusion, a comprehensive assessment of the biocompatibility of modified polysaccharides is of utmost importance. For the modified polysaccharides, their biocompatibility and safety are crucial for gaining public acceptance and meeting regulatory requirements, especially in key fields such as biomedicine and food, where systematic biological evaluations must be conducted to ensure their safety [255,256].

### 4.11. Critical Comparison of Modification Strategies Across Studies

Despite the general trends described above, the effects of structural modification are not always consistent across studies. For sulfation, some studies report that higher degrees of substitution correlate with stronger bioactivity [198], whereas others show that moderate substitution yields optimal effects and that excessive substitution may reduce activity [174]. These discrepancies may arise from differences in the starting polysaccharide structure, the distribution of modification sites, and the methods used to evaluate activity. Similarly, modification can either decrease molecular weight through chain degradation or increase it through cross-linking, depending on the reagent used and reaction conditions [64,159]. From a scalability perspective, most modification methods require toxic reagents (chlorosulfonic acid, pyridine) and multi-step purification, which limits their industrial applicability and raises safety concerns regarding residual reagents [195,246]. These concerns suggest that the use of certain chemical reagents to enhance biological activity may not necessarily improve safety. Some of these reagents are known to be toxic or corrosive, and residual reagents may pose potential health risks if not adequately removed. Therefore, the chemical reagents used for modification should be carefully selected, and cytotoxicity tests should be conducted to evaluate the safety of the modified products. These contradictions highlight that modification outcomes are highly dependent on the structural features initially determined by extraction, and that a unified framework linking starting structure, modification parameters, and resulting activity–safety–scalability profiles is still lacking. This gap reinforces the need for SAR-guided modification design rather than empirical trial-and-error.

## 5. Discussion

Throughout this review, polysaccharide structure serves as the connecting thread that links extraction, biological activity, and structural modification into a coherent framework. The central finding is that extraction method selection is not a neutral technical decision but may shape the structural identity and subsequent bioactivity of polysaccharides. Structural parameters, including molecular weight, monosaccharide composition, glycosidic linkages, branching degree, uronic acid content, and chain conformation, jointly influence bioactivity rather than acting independently. Chemical modification strategies informed by SAR knowledge may enable rational bioactivity optimization, bridging the gap between structural characterization and functional design.

Despite these general trends, contradictory findings exist across studies. For molecular weight, both low- and high-molecular-weight fractions have been reported to exhibit superior activity, depending on the polysaccharide source and target activity. For extraction methods, ultrasonic treatment has been reported to both increase and decrease molecular weight depending on power, duration, and polymer concentration. For structural modification, higher substitution degrees do not always translate into stronger activity; moderate substitution may yield optimal effects, and excessive substitution may reduce activity or narrow the safety window. These contradictions suggest that no single structural parameter can predict bioactivity across all polysaccharides, and that the optimal structural features are system-specific. They also point to more fundamental limitations in the current research landscape.

Current SAR studies remain largely confined to primary structural features, whereas the mechanism by which higher-order structures regulate bioactivity remains poorly understood. The internal relationship between chain conformation, aggregate morphology, and receptor recognition has not been fully elucidated. Cross-study comparison is further hindered by methodological heterogeneity, and a standardized reporting framework for structure–activity data is still lacking. Moreover, most plant-derived polysaccharide extracts are heterogeneous mixtures; the potential contribution of co-extracted proteins, polyphenols, and other contaminants is rarely characterized, which complicates the attribution of activity to specific structural features.

These limitations are compounded by several methodological problems. First, molecular weight determination depends on calibration standards that may not match the sample, leading to over- or underestimation. Second, NMR analysis of complex polysaccharides often produces overlapping signals that are difficult to assign. Third, SEM and AFM capture only dried or surface states, which may not reflect solution conformation. Fourth, most studies do not report the purity, protein content, polyphenol content, or molecular-weight distribution of polysaccharide extracts. Fifth, the safety and scalability of modified polysaccharides are rarely evaluated systematically; most modification methods require toxic reagents and multi-step purification, and residual reagent levels are seldom reported.

These methodological issues extend to the industrial application of extraction technologies. The environmental benefits of green extraction technologies should be evaluated critically rather than assumed. A fair comparison should consider yield, processing time, energy consumption, solvent usage, environmental impact, cost, and scalability simultaneously. Therefore, no single method is universally optimal; the choice depends on the target polysaccharide, the desired structural features, and the intended application. Life-cycle assessment and techno-economic analysis are needed for fair comparison across extraction technologies.

Addressing these challenges will require coordinated efforts on multiple fronts. Future research should prioritize: (1) standardized reporting of extraction conditions, structural characterization, purity and activity evaluation to enable cross-study comparison as much as possible; (2) application of advanced analytical techniques such as CD, SAXS, and NMR to resolve higher-order structures and their functional implications; (3) SAR-guided modification design rather than empirical trial-and-error, with systematic evaluation of activity, safety, stability, and scalability; (4) assessment of the physiological stability and biocompatibility of modified polysaccharides, including gastric acid stability, enzymatic stability, and residual reagent toxicity; and (5) integration of computational modeling and AI-assisted approaches to predict structure–activity relationships and guide the rational design of polysaccharide-based bioactives.

## 6. Conclusions and Perspectives

This review integrates extraction, structure–activity relationships (SAR), and structural modification of plant-derived polysaccharides into a unified framework. Extraction method selection may shape the structural identity and subsequent bioactivity of polysaccharides, and structural parameters appear to jointly influence bioactivity rather than acting independently. Chemical modification informed by SAR knowledge may enable rational bioactivity optimization, although contradictory findings, methodological limitations, and extract heterogeneity still hinder cross-study comparison. Future research could prioritize standardized reporting, advanced structural analysis, SAR-guided modification design, and systematic evaluation of safety, stability, and scalability, with the precise application of polysaccharides in targeted delivery, tissue repair, and functional materials as a promising direction.

## Figures and Tables

**Table 1:** Comparison of this review with recent related reviews.

Review	Year	Scope	Focus	Key Difference from the Present Review
Huang & Huang (2020) [19]	2020	Plant polysaccharides	Extraction, separation, modification, and antioxidant activity	Focuses on extraction, separation, modification, and antioxidant activity, but does not integrate these into a unified framework
Yang et al. (2022) [20]	2022	Polysaccharides from plants and fungi	Effect of structure and preparation method on bioactivity	Covers plants and fungi; discusses extraction, structure, and modification separately without integrating them into a unified framework
Li et al. (2022) [21]	2022	Land plant polysaccharides	Chemical modification, characterization, and activity changes	Modification discussed in isolation; extraction method selection and SAR knowledge are not linked
Zhang et al. (2025) [22]	2025	Edible mushroom polysaccharides	Structural characteristics, chemical modification, and SAR	Restricted to edible mushroom polysaccharides; extraction methods not systematically discussed
Hussain & Bokhari (2025) [14]	2025	Plant polysaccharides in diabetes mellitus	Extraction techniques, structural characterization, and mechanistic pathways	Disease-specific scope (diabetes); focuses on antidiabetic mechanisms rather than a generalized extraction–SAR–modification framework
Zhang et al. (2026) [18]	2026	Plant polysaccharides	Extraction methods, physicochemical properties, structures, and bioactivity	Does not cover structural modification, physiological stability, or safety evaluation
Zhao et al. (2026) [23]	2026	Polysaccharide extraction	Extraction mechanisms, hybrid strategies, and emerging technologies	Limited to extraction technologies; SAR and structural modification are not integrated
Present review	2026	Plant-derived polysaccharides	Extraction, SAR, and modification	Integrates all three aspects into a unified framework; proposes evidence-based method selection

**Table 2:** Yields of polysaccharides extracted by different techniques.

Polysaccharides	Extraction Method	Ratio of Powder to Solvent (g/mL)	Temperature	Extract Time	Extraction Yield	A Method for Obtaining a Higher Polysaccharide Yield	Reference
Polysaccharides from *Fragaria vesca* L. leaf	Cold alkaline extraction	1:10	Room temperature	24 h	2.5%	Ultrasound-assisted alkaline extraction	[25]
	Hot alkaline extraction	1:10	Boiling	70 min	7.7%		
	Ultrasound-assisted alkaline extraction	1:10	25 °C	40 min (60 W)	8.4%		
	Microwave-assisted alkaline extraction	1:10	80 °C	20 min (200 W)	6.7%		
Polysaccharides from *Panax ginseng*	Hot water extraction	1:40	90 °C	5 h	28.5 ± 1.62%	Microwave extraction	[26]
	Microwave-Assisted Extraction	1:30	70 °C	550 W microwave extraction for 6 min	41.6 ± 0.09%		
Polysaccharides from water lettuce (*Pistia stratiotes*) leaf	Ultrasound-assisted extraction	1:40	50 °C	180 W ultrasound for 3 h	9.16 ± 2.14%	Ultrasound-assisted extraction	[27]
	Hot water extraction	1:40	90 °C	8 h	4.41 ± 0.36%		
Polysaccharides from *Geum japonicum* var. *Chinense* F. Bolle	Heated Reflux Extraction	1:30	100 °C	2 h	4.49 ± 0.10%	Alkali-Assisted Extraction	[28]
	Acid-Assisted Extraction	1:30	60 °C	2 h	3.00 ± 0.02%		
	Alkali-Assisted Extraction	1:30	60 °C	2 h	14.21 ± 1.25%		
	Enzyme-Assisted Extraction	1:30	50 °C (enzyme incubation); 90 °C (enzyme inactivation)	2 h (enzyme incubation); 20 min (enzyme inactivation)	8.09 ± 0.24%		
	Microwave-Assisted Extraction	1:30	80 °C	480 W microwave power for 10 min	2.85 ± 0.02%		
	Pressurized Liquid Extraction	1:30	55 °C	Extract for 40 min at 1.6 MPa	6.28 ± 0.09%		
	Deep Eutectic Solvents Extraction	1:30	80 °C (Heated to a clear liquid at 80 °C and then extracted at 100 °C)	2 h	4.40 ± 0.16%		
Polysaccharides from maca roots	Hot water extraction	1:40	90 °C	3 h	13.08 ± 0.19%	Ultrasonic-assisted extraction (The solvent is a deep eutectic solvent)	[29]
	Enzyme-assisted extraction	1:40	50 °C (enzyme incubation); 100 °C (enzyme inactivation)	12 h (enzyme incubation); 5 min (enzyme inactivation)	6.22 ± 0.21%		
	Ultrasonic-assisted extraction (The solvent is water)	1:40	40 ± 2 °C	300 W for 20 min	15.16 ± 0.29%		
	Ultrasonic-assisted extraction (The solvent is a deep eutectic solvent)	1:40	40 ± 2 °C	300 W for 20 min	30.85 ± 0.37%		
Polysaccharides from Young Hulless Barley Leaf	Hot water extraction	1:25	80 °C	2 h	4.11 ± 0.38%	Combined enzymatic method	[30]
	High-pressure steam extraction	1:25	121 °C (liquid cycle (235 Kpa); 80 °C (water extraction)	30 min (liquid cycle 235 Kpa); 2 h (water extraction)	18% ~ 20%		
	Alkali-Assisted Extraction	1:25	80 °C	2 h	18% ~ 20%		
	Xylanase Extraction	1:25	50 °C; pH5.0	3 h	18% ~ 20%		
	Cellulase-assisted extraction	1:25	50 °C; pH5.0	3 h	18% ~ 20%		
	Combined extraction of xylanase and cellulase	1:25	50 °C; pH5.0	3 h	22.63 ± 0.22%		

**Table 3:** The influence of extraction methods on structure and activity.

Extraction Method	Effect on Molecular Weight	Effect on Conformation	Key Trade-Off	Applicable Scenarios	Activity Indication
Hot water extraction	Bidirectional adjustment	Helix disruption	High scalability but may degrade higher-order structure	Low cost, industrial scale-up	Suitable for activities not relying on higher-order structure
Microwave/Ultrasound-assisted extraction	Controlled reduction	Shear effect	High yield but may reduce molecular weight	Quick extraction, molecular weight tailoring	Activity can be modulated by adjusting intensity
Enzyme-assisted extraction	Bidirectional adjustment	Respects natural conformation	Preserves structure but high enzyme cost	Requires activities with higher-order structure research	Respects receptor binding ability
Cooperative extraction	Tunable	Tunable	Flexible but scalability unverified	Optimization of yield and structure	Flexible to adapt to different activity requirements

**Table 4:** The molecular weights of different components of polysaccharides vary and affect their activity.

Polysaccharide	Abbreviations for Different Polysaccharide Components	Molecular Weight	More Active Component	The Molecular Weight Range with Better Inter-Component Activity (Described as Low, Medium, and High)	Reference
*Artemisia argyi* polysaccharides	AAP-1	89.682 kDa	AAP-5 exhibits better in vitro antioxidant activity	lower molecular weight	[107]
	AAP-3	45.848 kDa			
	AAP-5	25.934 kDa			
*Cuminum cyminum* Polysaccharides	CCP1	512 KD	CCP2 has high immunological activity	lower molecular weight	[108]
	CCP2	206.9 KD			
Litchi polysaccharides	LP1	378.67 KDa	LP2 has better immunomodulatory and prebiotic activity	lower molecular weight	[109]
	LP2	16.96 KDa			
*Codonopsis* polysaccharides	CPPS-I	570 kDa	The anti-tumor and immunomodulatory activities of CPPS-II were enhanced	medium molecular weight	[110]
	CPPS-II	980 kDa			
	CPPS-III	3300 kD			
*Cucurbita Moschata* (Butternut Squash) Polysaccharides	NJBTF1	90 kDa	NJBTF1 showed the strongest immune activity	higher molecular weight	[111]
	NJBTF2	31 kDa			
	NJBTF3	19 kDa			
*Sagittaria sagittifolia* L. polysaccharides	SPC-60	52.0 kDa	The SPC-70 exhibits a stronger ability to scavenge DPPH, ABTS and hydroxyl radicals.	higher molecular weight	[112]
	SPC-90	229.4 kDa			
	SPC-80	230.6 kDa			
	SPC-70	294.9 kDa			

**Table 6:** The structural modification of polysaccharides and its impact on their biological activity.

Modification Methods	Polysaccharide	Modified Result	Reference
Sulfation	Dandelion root polysaccharides	Enhanced antioxidant and hypoglycemic activity, improving the chelation ability of Fe^2+^.	[173]
	*Sagittaria trifolia* polysaccharide	Antioxidant activity altered, procoagulant activity transformed into anticoagulant activity	[174]
	*Arnebia euchroma* (Royle) Johnst. *Roots* polysaccharide	The conformation changes to a triple helix structure, and both the antioxidant activity and immunomodulation are enhanced	[175]
	Sulfated alfalfa polysaccharidesn	The antioxidant activity is enhanced, effectively increasing the survival rate of intestinal epithelial cells that have been subjected to oxidative stress.	[176]
Phosphorylation	*Astragalus* polysaccharide	Enhanced activity against duck hepatitis A virus	[177]
	Polysaccharide from *Asarum sieboldii* Miq	A triple-helix conformation is formed; The scavenging capacity for DPPH and ABTS radicals is enhanced.	[178]
Selenizing	*Astragalus* polysaccharide	Enhanced antioxidant activity	[159]
	*Codonopsis pilosula* polysaccharide	Possesses strong selenium release capacity, enhancing antioxidant activity	[179]
	Tea polysaccharides	Both antioxidant activity and hypoglycemic activity were enhanced.	[180]
Carboxymethylation	Corn bran polysaccharide	Exceptional antioxidant capacity and potent anti-tumor activity	[181]
	Guar gum from *Cyamopsis tetragonoloba*	Excellent antioxidant and antibacterial activity	[182]
	*Gastrodia elata* polysaccharide	Enhanced the scavenging efficacy against DPPH free radicals, hydroxyl free radicals and superoxide anions	[183]
	*Chaenomeles sinensis* polysaccharide	Significant enhancement of anti-inflammatory and antioxidant responses	[184]
Methylation	Soy hull polysaccharides	Enhanced polysaccharide emulsifying capacity	[185]
Acetylate	Mung bean peel polysaccharide	The structure has changed and the free radical scavenging ability of hydroxyl groups has been enhanced	[186]
	*Dendrobium officinale* polysaccharides	Boost Immunity	[187]
	Polysaccharides from *Rhododendron dauricum* leaves	The triple-helix conformation disappears, enhancing anti-complementary activity.	[188]
	polysaccharide from *Houttuynia cordata* rhizome	Increased solubility, with enhanced α-glucosidase inhibitory activity to a certain extent	[189]
Complexing with metal ions	*Glycyrrhiza* polysaccharide	From an irregular porous structure to a spherical porous structure, it exhibits excellent antioxidant and anti-inflammatory properties and promotes wound healing	[190]
	*Dioscorea oppositifolia* L. peel polysaccharides	The antioxidant activity is enhanced, and it can be used as a multifunctional iron supplement	[191]
	Gum Arabic *Acacia senegal* and *Acacia seyal* trees	Modulation of apoptosis-related proteins significantly reduced the survival rates of A549 and HEK239T cells.	[192]
	*Lepidium meyenii* (maca) Polysaccharide	Enhance the free radical scavenging activity against DPPH and ABTS, and the proliferation activity of anti-cancer cells	[193]
	Ginger peel polysaccharides	Thermal stability is enhanced, and anti-inflammatory activity is increased.	[194]

**Table 7:** A strategy for selecting structural modification methods.

Modifying Methods	Introduction of Functional Groups	Principal Structural Parameters Changed	Verified Structure–Activity Relationship	Preferred Application Scenarios
Sulfation	-SO_3_H	Negative charge density	Charge → Antioxidant/Anticoagulant/Antiviral	Requires high negative charge density for activity
Phosphorylation	-PO_3_H_2_	Chain conformation, water solubility	Configuration → Immune/Antioxidant	Requires induction of helical conformation of polysaccharides
Carboxymethylation	-CH_2_COOH	Water solubility, surface morphology	Dissolution → Bioavailability → Activity Requires	natural poor-solubility polysaccharides
Selenation	-Se	Particle size, surface morphology	Trace element synergy → Dual activity	Enhances antioxidant/immune/anti-aging
Methylation	-OCH_3_	Surface hydrophobicity	Hydrophobicity balance → Interface activity	Requires emulsifying ability of polysaccharides
Acetylation	-COCH_3_	Bi-polarity, conformation	Amphiphilicity → Solubility → Immunity	Improving solubility and immunological activity
Metal ion complexation	Fe^2+^/Zn^2+^, etc.	Morphology, molecular weight	Morphology structure → Physicochemical properties → Activity	Increased activity and the need to supplement trace elements

## Data Availability

Data sharing not applicable to this article as no datasets were generated or analyzed during the current study.

## Abbreviations

The following abbreviations are used in this manuscript:

SAR	Structure–activity relationship
MAE	Microwave-assisted extraction
EAE	Enzyme-assisted extraction
HWE	Hot water extraction
UAE	Ultrasound-assisted extraction
UEAE	Ultrasonic-enzymatic-assisted extraction
DES	Deep eutectic solvent
ATPS	Aqueous two-phase systems
CPPS	*Codonopsis pilosula* polysaccharides
LP	polysaccharides of *Longan Pulp*
RIP	Polysaccharide components from *Roots of Isatis indigotica Fort*
Ara	Arabinose
Fuc	Fucose
Fru	Fructose
Gal	Galactose
Glc	Glucose
Man	Mannose
Rha	Rhamnose
Xyl	Xylose
LLp	*Ligustrum lucidum* flower polysaccharides
APS	*Astragalus* polysaccharides
LBP	polysaccharide from Lycium barbarum Fruits
MD	Molecular dynamics
DM	Degree of methyl-esterification
HG	Homogalacturonan
RG-I	Rhamnogalacturonan-I
RG-II	Rhamnogalacturonan-II
XGA	Xylogalacturonan
GalA	Galacturonic acid
SEM	Scanning electron microscopy
AFM	Atomic force microscopy
PST	Polysaccharides from *Sagittaria trifolia*
ASP	polysaccharide from *Asarum sieboldii* Miq
GPs	*Ginger* peel polysaccharides
DS	Degree of substitution
CD	Circular dichroism
SAXS	Small angle X ray scattering
NMR	Nuclear magnetic resonance
NJBTF	*Cucurbita Moschata (Butternut Squash)* Polysaccharides
CCP	*Cuminum cyminum Polysaccharides* Polysaccharides
HNPCE	Homogenization-assisted negative pressure cavitation extraction
AX	Arabinoxylans
